# Soft, Dynamic Hydrogel Confinement Improves Kidney Organoid Lumen Morphology and Reduces Epithelial–Mesenchymal Transition in Culture

**DOI:** 10.1002/advs.202200543

**Published:** 2022-05-14

**Authors:** Floor A. A. Ruiter, Francis L. C. Morgan, Nadia Roumans, Anika Schumacher, Gisela G. Slaats, Lorenzo Moroni, Vanessa L. S. LaPointe, Matthew B. Baker

**Affiliations:** ^1^ MERLN Institute for Technology‐Inspired Regenerative Medicine Department of Complex Tissue Engineering Maastricht University Universiteitssingel 40 Maastricht 6229 ER the Netherlands; ^2^ MERLN Institute for Technology‐Inspired Regenerative Medicine Department of Cell Biology‐Inspired Tissue Engineering Maastricht University Universiteitssingel 40 Maastricht 6229 ER the Netherlands; ^3^ Department II of Internal Medicine and Center for Molecular Medicine Cologne University of Cologne, Faculty of Medicine and University Hospital Cologne Cologne 50937 Germany; ^4^ Cologne Excellence Cluster on Cellular Stress Responses in Aging‐Associated Diseases (CECAD) University of Cologne Faculty of Medicine and University Hospital Cologne Cologne 50931 Germany

**Keywords:** dynamic hydrogels, epithelial–mesenchymal transition, kidney organoids, primary cilia, viscoelastic

## Abstract

Pluripotent stem cell‐derived kidney organoids offer a promising solution to renal failure, yet current organoid protocols often lead to off‐target cells and phenotypic alterations, preventing maturity. Here, various dynamic hydrogel architectures are created, conferring a controlled and biomimetic environment for organoid encapsulation. How hydrogel stiffness and stress relaxation affect renal phenotype and undesired fibrotic markers are investigated. The authors observe that stiff hydrogel encapsulation leads to an absence of certain renal cell types and signs of an epithelial–mesenchymal transition (EMT), whereas encapsulation in soft, stress‐relaxing hydrogels leads to all major renal segments, fewer fibrosis or EMT associated proteins, apical proximal tubule polarization, and primary cilia formation, representing a significant improvement over current approaches to culture kidney organoids. The findings show that engineering hydrogel mechanics and dynamics have a decided benefit for organoid culture. These structure–property–function relationships can enable the rational design of materials, bringing us closer to functional engraftments and disease‐modeling applications.

## Graphical abstract

Current kidney organoid protocols often lead to off‐target cells and phenotypic alterations. Various dynamic hydrogel architectures are investigated for organoid encapsulation. Encapsulation in soft, fast stress‐relaxing hydrogels leads to all major renal segments, fewer fibrosis or epithelial‐mesenchymal transition (EMT) associated proteins, apical proximal tubule polarization, and primary cilia formation, representing a significant improvement over current approaches to culture kidney organoids.

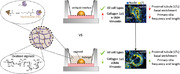

## 1 Introduction

Within the organoid field, various hydrogels have been investigated to influence cell behavior,^[^ ^1^ ^]^ including biopolymer‐based hydrogels (e.g., alginate),^[^ ^2^ ^]^ fully synthetic materials (polyethylene glycol (PEG) and polyacrylamide),^[^ ^3^ ^]^ and bio hybrids (Matrigel combined with PEG, fibrin, or alginate).^[^ ^4^ ^]^ However, these hydrogels were mainly used in intestinal,^[^ ^5^ ^]^ pancreatic,^[^ ^6^ ^]^ neural,^[^ ^7^ ^]^ and hepatic^[^ ^8^ ^]^ organoid cultures, while few have been applied in kidney organoid culture.^[^ ^3^, ^9^ ^]^ Moreover, existing synthetic hydrogels for organoid culture largely rely on covalent or non‐reversible cross‐linking interactions, while the natural extracellular matrix (ECM) is dynamic.^[^ ^10^ ^]^ Dynamic covalent cross‐linked hydrogels have been of increased interest in the biomaterials field, as they allow recapitulation of both stiffness and dynamic stress‐relaxing character of the native ECM.^[^ ^10^, ^11^ ^]^ To date, dynamic hydrogels have been observed to influence cell fate when single cells were encapsulated, as observed with extended motor neurons axon bodies,^[^ ^12^ ^]^ 3D cell spreading and focal adhesion of human mesenchymal stem cells,^[^ ^13^ ^]^ and increased cartilage matrix formation by chondrocytes,^[^ ^14^ ^]^ but their application in aggregate or organoid culture is less studied.

Kidney organoids derived from induced pluripotent stem cells (iPSCs) mimic the organogenesis of the human kidney.^[^ ^15^ ^]^ In addition to their potential for studying development, disease modeling, and drug screening, kidney organoids can be transplanted as a functional graft^[^ ^16^ ^]^ in patients with chronic kidney disease, which affects 11–13% of the population worldwide.^[^ ^17^ ^]^ Nevertheless, there are still many challenges to overcome before organoids are suitable for widespread clinical application. For example, current kidney organoids resemble an immature developing kidney at both the transcriptional^[^ ^18^ ^]^ and morphological level,^[^ ^19^ ^]^ and prolonged culture does not improve their maturation. Moreover, morphological changes, an increase in off‐target cell populations,^[^ ^18^ ^]^ aberrant ECM, containing increased types I and VI collagen and alpha smooth muscle actin (aSMA) expression are observed.^[^ ^9^ ^]^ We have previously shown a reduction in the onset of the aberrant ECM expression and off‐target cell populations by encapsulating the organoids in a soft thiol‐ene cross‐linked alginate hydrogel.^[^ ^9^ ^]^ This initial study showed the importance of the surrounding environment on the organoid ECM, which led us to wonder how the mechanical stiffness and hydrogel dynamics of the encapsulating matrix can affect organoids.

**Figure 1:**
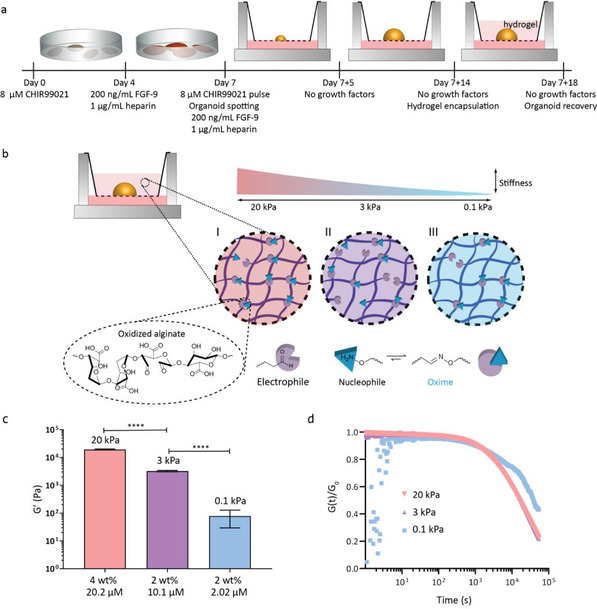
Design of hydrogels of varying stiffness. a) Overview of iPSC differentiation and organoid generation. iPSCs are differentiated for 7 days, after which they are grown and matured as organoids for 14 days on the air–liquid interface (day 7+14). Organoids are then encapsulated in the different hydrogels (or left on the air–liquid interface as a control) and cultured for 4 subsequent days until day 7+18. b) Schematic of the different hydrogel systems. All hydrogels are based on oxidized alginate cross‐linked with oxime. Increasing the percentage (by weight) of oxidized alginate resulted in increased entanglement and binding sites, and therefore increased stiffness. Similarly, an increase in oxime increased the cross‐linking density, and therefore increased the stiffness of the hydrogel. c) The shear moduli (G′) of the three different hydrogel compositions (*N* = 3, error bars representing standard deviation), of 20, 3, and 0.1 kPa for the 4% alginate‐20.2 µM oxime; 2% alginate‐10.1 µM oxime, and 2% alginate‐2.02 µM oxime hydrogels, respectively. The hydrogels had significantly different stiffnesses (one‐way ANOVA, *p* < 0.0001). d) The stress relaxation of the different hydrogels compositions is similar (one‐way ANOVA, *p* = 0.32), with *t*_1/2_ values from 1.6–3.9·10^4^ s, despite their different stiffness.

Here, we tested the influence of dynamic hydrogels on kidney organoids by designing a small hydrogel library based on oxidized alginate: three hydrogels of tuneable stiffness (ranging from 0.1 to 20 kPa), and two soft hydrogels (0.1 kPa) with different stress relaxation (slow and fast). We used an imine‐type dynamic covalent cross‐linking possessing a range of equilibrium constants (*K*_eq_) that affect the hydrogel stiffness and tuneable hydrolysis rates (*k*_−1_) that change the rate of cross‐link rearrangement and stress relaxation.^[^ ^20^ ^]^ Kidney organoids cultured until day 7+14 (namely, 7 days of iPSC differentiation and 14 days of organoid culture on an air–liquid interface) were encapsulated in these hydrogels and cultured for 4 subsequent days (**Figure** **1**a). We confirmed the formation of renal structures by immunohistochemistry. We investigated the expression of off‐target ECM and epithelial–mesenchymal transition (EMT)‐related markers, as well as variations in the formation of lumen and cilia structures in organoids cultured in hydrogels of different stiffness and stress relaxation. Encapsulation in soft hydrogels with fast‐relaxation properties resulted in more mature kidney organoids, determined by the above parameters, and compared to the stiffer hydrogels or slow‐relaxing hydrogels. Our findings reinforce the concept of carefully selecting not only the stiffness, but also stress relaxation properties of encapsulating matrices and highlight the potential of tuning hydrogel properties to influence organoid cultures.

## 2 Results and Discussion

### 2.1 Hydrogels were Designed with Varying Stiffness

To determine the role of hydrogel stiffness on renal organoid phenotype and ECM deposition, we designed three alginate hydrogels with varying stiffness (Figure 1b). We selected sodium alginate, a naturally‐derived, biocompatible, and non‐adhesive biomaterial, which is biodegradable when oxidized, allows free diffusion in low wt% conc. hydrogels^[^ ^21^ ^]^ and have been used in Food and Drug Administration‐approved applications.^[^ ^22^ ^]^ Sodium alginate can form an ECM‐like hydrogel and has previously been shown to support culture of kidney organoids.^[^ ^9^ ^]^ We oxidized the alginate to obtain aldehyde groups for imine‐type cross‐linking, which allows for dynamic reshuffling of the cross‐links in cell culture conditions.^[^ ^20^ ^]^

Sodium alginate was oxidized using sodium periodate (NaIO_4_) at a 10% theoretical degree of oxidation, then characterized by proton nuclear magnetic resonance (^1^H‐NMR, Figure S1, Supporting Information) and gel permeation chromatography (GPC; Figure S2 and Table S1, Supporting Information). As a cross‐linker, we used a small bifunctional oxime (O,O′‐1,3‐propanediylbishydroxylamine). Starting at 2 wt% oxidized alginate, increasing the concentration of bifunctional oxime increased the cross‐linking density, which subsequently resulted in an increased stiffness, changing from 0.1–3.0 kPa for 2.02 to 10.1 µM of oxime cross‐linker, respectively (Figure 1b,c and Figure S3a–c, Supporting Information). To obtain a higher stiffness, we increased the weight percentage of the oxidized alginate (from 2 to 4 wt%) while keeping the 1:1 oxime to aldehyde ratio (20.2 µM oxime cross‐linker), which resulted in a hydrogel with a stiffness of 20 kPa (Figure 1c and Figure S3a–c, Supporting Information). While the stiffness of the hydrogels was significantly different in each composition (*p* < 0.0001), the characteristic stress relaxation times (*t*_1/2_, defined as the time taken for the relaxation modulus to reach half of its initial value) were similarly long, with values from 1.6–3.9·10^4^ s (*p* = 0.32, Figure 1d).

### 2.2 Kidney Organoids Formed in all Hydrogels but Selective Renal Cell Types were Absent in the 20 kPa Hydrogel

Kidney organoids were cultured until day 7+14, at which point they were encapsulated in the different hydrogels for 4 additional days of culture (Figure 1a). The starting point of day 7+14 was selected because our previous work demonstrated that an overexpression of type 1a1 collagen began at day 7+14 of organoid culture and could be significantly reduced by encapsulating the organoids in hydrogels for 4 additional days.^[^ ^9^ ^]^ The hydrogel solutions were added on top of the organoid on the air–liquid interface, and hydrogel cross‐linking was observed after approximately 1 h incubation at 37 °C. After 4 d (day 7+18), the hydrogels were removed, and organoids were stained with calcein AM and EthD‐1 to assess live/dead cells (Figure S3e, Supporting Information). We detected no difference in the morphology of the organoids (bright‐field microscopy, Figure S4a, Supporting Information) or live/dead cells compared to organoids cultured on the air–liquid interface at day 7+18 (Figure S4b, Supporting Information).

**Figure 2:**
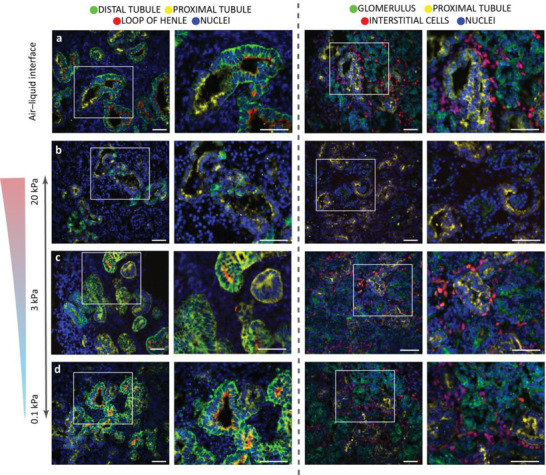
Kidney organoids formed in all dynamic hydrogels, but a subset of renal cell types is absent in the 20 kPa hydrogel. Culture of the organoids for 4 days (from day 7+14 to 7+18) in hydrogels did not affect the presence of nephron segments in the 0.1 kPa (d) or 3 kPa (c) hydrogels when compared to organoids grown on an air–liquid interface until day 7+18 (a). Only the organoids encapsulated in the stiffest 20 kPa hydrogel (b) lacked loop of Henle segments (two left columns) and interstitial cells (two right columns). Staining for distal tubules (E‐cadherin; ECAD); the loop of Henle (NKCC2; SLC12A1); proximal tubules (lotus tetragonolobus lectin; LTL); glomeruli (nephrin; NPHS1); and interstitial cells (homeobox protein Meis 1/2/3; MEIS1/2/3) as indicated. DAPI staining (blue) for nuclei. The white box denotes the area of interest enlarged in the respective right panel. Scale bars: 50 µm. Representative images of *N* = 3 organoid batches with *n* = 3 organoids per batch. See single channels in Figure S5, Supporting Information.

To determine the effect of the hydrogel stiffness on the presence of different renal segments, the organoids were stained with relevant markers for distal tubules (E‐cadherin; ECAD), glomeruli (nephrin; NPHS1), interstitial cells (homeobox protein Meis 1/2/3; MEIS1/2/3), loop of Henle (NKCC2; SLC12A1), and proximal tubules (LTL). The organoids recovered from the 0.1 and 3 kPa hydrogel possessed all expected segments (**Figure** **2**c,d and Figures S5c,d and S6a, Supporting Information) and had no distinguishable differences from organoids cultured on the air–liquid interface (Figure 2a, Figures S5a and S6a, Supporting Information). However, organoids encapsulated in the stiffer 20 kPa hydrogel lacked interstitial (*p* < 0.0001) and loop of Henle cells, and showed significantly diminished lumen structures (LTL, *p* = 0.0034 and *p* = 0.0461), glomeruli (nephrin, *p* < 0.0001 and *p* = 0.0087) and distal tubules (ECAD, *p* = 0.0012; *p* = 0.0005 and *p* = 0.0316) (Figure 2b, Figures S5b and S6a, Supporting Information) compared to organoids cultured on both the air–liquid interface and other hydrogels (0.1 and 3 kPa, respectively).

### 2.3 No EMT Observed in Organoids Encapsulated in the Soft 0.1 kPa Hydrogels

Because previous work demonstrated that prolonged (beyond day 7+14) organoid culture led to protein expression indicating fibrosis, namely collagens 1a1 and 6a1,^[^ ^9^ ^]^ we wished to determine whether the kidney organoids encapsulated in the hydrogels showed fibrotic markers. Immunohistochemistry data showed a significant reduction of type 1a1 collagen (Figures S6b and S7b–d, Supporting Information) in organoids encapsulated in all hydrogels compared to those cultured on the air–liquid interface control (Figures S6b and S7a, Supporting Information, 20 kPa; *p* = 0.0039, 3 kPa; *p* = 0.016 and 0.1 kPa; *p* < 0.0001). In contrast, the expression of type 6a1 collagen mostly unchanged, with only 3 kPa showing a slight reduction in type 6a1 collagen (Figure S6b, *p* = 0.015). This indicates that the hydrogel stiffness had a more selective modifying effect on the type 1a1 collagen. This selective effect has been observed previously when kidney organoids were encapsulated in a soft (0.2 kPa), thiol‐ene cross‐linked, alginate hydrogel,^[^ ^9^ ^]^ and suggests a reduced fibrotic phenotype when organoids are encapsulated in soft hydrogels.

**Figure 3:**
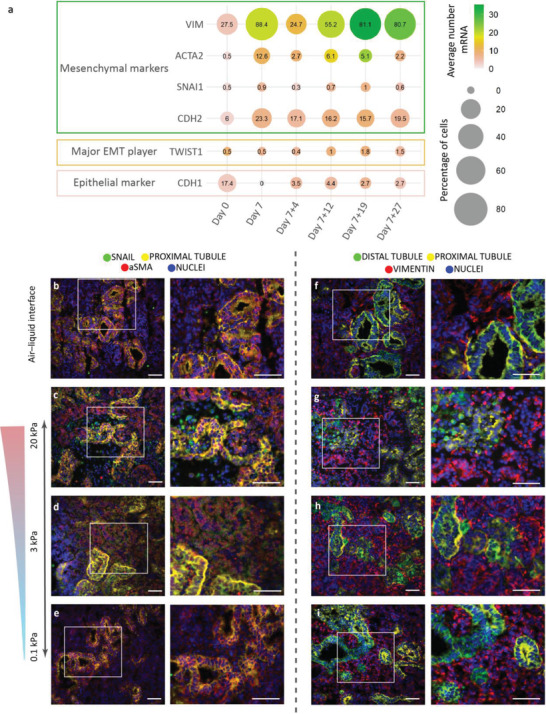
Evidence of EMT observed in organoids encapsulated in the 3 and 20 kPa hydrogels. a) Analyzing single‐cell RNA sequencing datasets from the literature^[^ ^18^ ^]^ showed an increased number (%) of cells that expressed the EMT‐related marker (*TWIST1*) and mesenchymal markers (*VIM, ACTA2, SNAI1*, and *CDH2*) and a reduced number (%) of cells with the epithelial marker (*CDH1;* more information see Supporting Information). b–i) Encapsulated organoids until day 7+18 showed an expression of the EMT marker SNAIL (green, two left columns) in the 20 kPa (c) and 3 kPa (d) hydrogels, whereas no expression is detected in the 0.1 kPa hydrogel (e) or the air–liquid interface control (b). Moreover, higher expression of the mesenchymal marker vimentin (red, two right columns) is observed in the 20 kPa (g) and 3 kPa (h) hydrogels compared to the air–liquid interface (f). Staining for distal tubules (E‐cadherin: ECAD), proximal tubules (lotus tetragonolobus lectin: LTL), and fibroblasts (alpha smooth muscle actin: *α*SMA) as indicated. DAPI staining (blue) for nuclei. The white boxes denote the areas of interest enlarged in the respective right panels. Scale bars: 50 µm. Representative images of *N* = 3 organoid batches with *n* = 3 organoids per batch. See single channels in Figure S7, Supporting Information.

Since EMT is an early marker of renal fibrosis,^[^ ^23^ ^]^ we also analyzed the expression of EMT‐related markers to determine the influence of hydrogel stiffness. We began by analyzing an existing single‐cell RNA sequencing dataset^[^ ^18^ ^]^ from kidney organoids cultured up to day 7+27^[^ ^18^ ^]^ for EMT‐related markers. We found the following indicators of an EMT: the upregulation of twist family bHLH transcription factor 1 (*TWIST1*), snail zinc‐finger transcriptional factor 1 (*SNAI1* encoding SNAIL), N‐cadherin (*CDH2*), aSMA (*ACTA2*), and vimentin (*VIM*), and the downregulation of E‐cadherin (*CDH1*) (**Figure** **3**a). These EMT markers were deregulated from day 7+12 up until day 7+27 on the air–liquid interface, especially the mesenchymal marker vimentin, while the epithelial marker E‐cadherin was downregulated (Figure 3a and Figure S8, Supporting Information).

For organoids cultured in the hydrogels up to day 7+18, immunohistochemistry showed the classic EMT markers in the 3 and 20 kPa hydrogels. SNAIL‐positive cells were found in organoids cultured in the 3 kPa (Figure 3d and Figure S6c, Supporting Information) and significantly increased in the 20 kPa (Figure 3c and Figure S6c, Supporting Information, *p* < 0.0001) hydrogels, but were not observed in the 0.1 kPa hydrogel (Figure 3e and Figure S6c, Supporting Information) or on the air–liquid interface (Figure 3b and Figure S6c, Supporting Information). Organoids cultured in the two stiffer hydrogels (3 and 20 kPa; Figure 3d,c) expressed significantly more aSMA than organoids cultured in the 0.1 kPa hydrogel (Figure 3e and Figure S6c, Supporting Information, *p* = 0.021 for 3 kPa and *p* < 0.0001 for 20 kPa). Expression of the mesenchymal marker vimentin was observed in all organoids (Figure 3f–i), with higher expression in the organoids in the 3 and 20 kPa hydrogels and air–liquid interface (Figure 3f,g) compared to the soft hydrogel (0.1 kPa, Figure 3i and Figure S6c, Supporting Information; *p* = 0.0035 for air–liquid interface and *p* = 0.0046 for 3 kPa). Vimentin expression establishes the presence of mesenchymal cells and is required for EMT‐related renal fibrosis,^[^ ^23^, ^24^ ^]^ where it is upregulated in proximal tubular cells when repair is initiated after epithelial injury.^[^ ^25^ ^]^ In all organoids, vimentin did not co‐stain with the proximal tubule or distal tubule cells, but was located between those segments (Figure 3f,i and Figure S9f–i, Supporting Information). These findings, together with the absence of interstitial (MEIS1/2/3^+^) cells, (Figure 2b and Figure S6a, Supporting Information), increased aSMA‐positive cells (Figure 3c and Figure S6c, Supporting Information), reduced lumen structures (Figures S5b, S6a, and S10c, Supporting Information), and diminished distal tubules (Figure 2b and Figure S6a, Supporting Information), in the stiffest 20 kPa hydrogel, suggested some epithelial cells have turned to mesenchymal cells and were contributing to the organoid stroma. While the mass of the hydrogel could also be hypothesized to modulate this effect, the mass of hydrogels applied to each organoid is identical.

Ondeck et al.^[^ ^26^ ^]^ saw a similar trend of increased mesenchymal markers, while losing epithelial characteristics when dynamically increasing the stiffness of a methacrylate glycosaminoglycan hyaluronic acid hydrogel from 0.1 to 3000 Pa. Others have reported that a softer environment can accelerate the differentiation of iPSC‐derived kidney organoids^[^ ^3b^ ^]^ and can prime undifferentiated cells to lineage commitment.^[^ ^27^ ^]^ Moreover, Chen et al.^[^ ^28^ ^]^ observed the prevention of transforming growth factor beta (TGF‐*β*1)‐induced EMT when porcine kidney proximal tubule cells were cultured on collagen type 1‐coated polyacrylamide gels of ≈0.2 kPa stiffness, while cells on a stiffer matrix (>0.7 kPa) highly expressed mesenchymal markers. Similarly, we observed the EMT marker SNAIL in the 3 and 20 kPa hydrogels but not in the 0.1 kPa hydrogel. These findings show increased evidence that a softer hydrogel correlates a reduced EMT response. Moreover, when looking at biological stiffness of an adult kidney (5–10 kPa),^[^ ^29^ ^]^ we would expect the hydrogels of 3 kPa to show the best outcome. However, we observed much better results for the soft hydrogel of 0.1 kPa, a stiffness more relevant to the developing embryonic kidney of <1 kPa. This observation argues for the importance of considering the embryonic environment when selecting a biomaterial's mechanical properties.

### 2.4 A Fast‐Relaxing Dynamic Hydrogel Further Reduced the Undesired EMT‐Related Marker Expression Pattern

**Figure 4:**
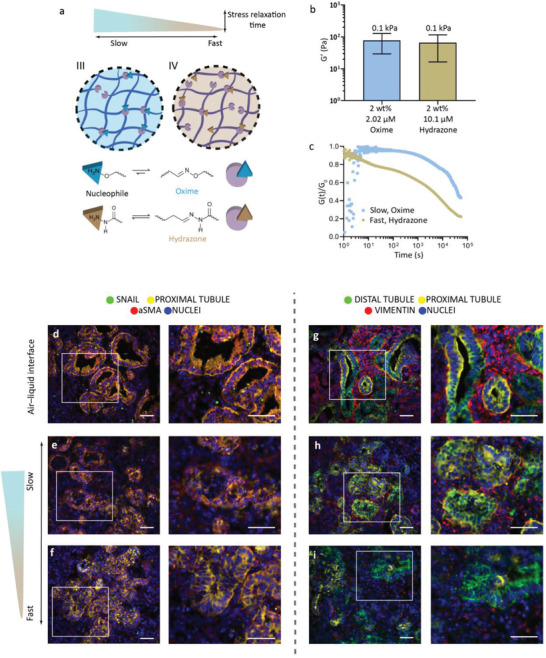
Fast‐relaxing dynamic hydrogel further reduced the undesired EMT‐associated markers. a) Schematic of two alginate hydrogels with different cross‐linkers—hydrazone or oxime—to influence stress relaxation times. b) The stiffness of both hydrogels is approximately 0.1 kPa (0.08 ± 0.05 and 0.07 ± 0.05 kPa for 2.02 µM oxime and 10.1 µM hydrazone, respectively; error bars represent the mean ± SD, *t*‐test, *p* < 0.0001). c) The stress relaxation time of the fast‐relaxing hydrazone hydrogel is an order of magnitude faster, with a value of 4.1·10^3^ s compared to the 3.9·10^4^ s for the soft 0.1 kPa oxime hydrogel (d–i). Encapsulated organoids until day 7+18 in the fast stress‐relaxing hydrogel (f,i) showed less staining for aSMA (red, two left columns) and vimentin (red, two right columns) compared to those grown in the slow‐relaxing hydrogel (e,h) and on the air–liquid interface (d,g). Staining for distal tubules (E‐cadherin: ECAD), proximal tubules (lotus tetragonolobus lectin: LTL), and the mesenchymal markers (Zinc finger protein SNAI1: SNAIL and Vimentin) as indicated. DAPI staining (blue) for nuclei. The white box denotes the area of interest enlarged in the respective right panel. Scale bars: 50 µm. Representative images of *N* = 3 organoid batches with *n* = 3 organoids per batch. See single channels in Figure S12, Supporting Information.

Besides stiffness (expressed as the shear moduli), stress relaxation plays a role in the cellular response to its surrounding material.^[^ ^13^, ^20^, ^30^ ^]^ We had so far kept the stress relaxation of the hydrogel similar (Figure 1d) to compare only the effect of stiffness (Figure 1c). To examine the effect of stress relaxation, we designed a fourth hydrogel with a similar stiffness as the soft oxime cross‐linked hydrogel (≈0.1 kPa, **Figure** **4**a,b) but with a faster stress relaxation time by using hydrazone as the cross‐linker (Figure 4a). The imine group hydrolysis rate (*k*_−1_) can be tuned by changing the electronegativity of the alpha group to the primary amine.^[^ ^20^ ^]^ In this case, we decreased the electronegativity of the alpha group by using hydrazone (adipic dihydrazide) instead of oxime to form a faster stress‐relaxing hydrogel (Figure 4a). A higher concentration of the cross‐linker hydrazone (10.1 µM) with 2 wt% oxidized alginate was used to obtain a hydrogel of similar stiffness (0.1 kPa) as the soft oxime hydrogel (Figure 4b and Figure S11a,b, Supporting Information). The characteristic stress relaxation time of the resulting hydrazone cross‐linked hydrogel was an order of magnitude faster, with a *t*_1/2_ value of 4.1·10^3^ s, compared to the 3.9·10^4^ s for the soft 0.1 kPa oxime cross‐linked hydrogel (Figure 4c).

Organoids were encapsulated in the soft and fast stress‐relaxing hydrogel at day 7+14 of culture and were cultured until day 7+18. We detected no difference in the morphology of the organoids (bright‐field imaging, Figure S11d, Supporting Information) or the ratio of live/dead cells compared to organoids cultured on the air–liquid interface (Figure S11e, Supporting Information). Recovered organoids showed all nephron segments in similar levels to organoids cultured on the air–liquid interface (Figures S6d and S12a,d, Supporting Information). A decrease in type 1a1 collagen expression was observed in organoids cultured in the soft, fast‐relaxing hydrazone hydrogel (Figures S6e and S12e, Supporting Information, *p* < 0.0001) similar to those cultured in the soft, slow‐relaxing oxime hydrogel (Figure S7a, Supporting Information), while no change in type 6a1 collagen expression was observed (Figures S6e and S12f, Supporting Information) compared to culture on the air–liquid interface (Figure S7a, Supporting Information). Moreover, there was less aSMA, indicating a reduction of myofibroblasts (Figure S6c, Supporting Information; *p* = 0.0006), and no SNAIL expression in the organoids encapsulated in the fast‐relaxing hydrazone hydrogels (Figure 4f) compared to the organoids cultured on the air–liquid interface (Figure 4d). Interestingly, lower expression of vimentin was observed in the organoids encapsulated in the fast‐relaxing hydrogel (Figure 4i and Figure S6f, Supporting Information; *p* < 0.0001) compared to those in the slow stress‐relaxing hydrogel (Figure 4h and Figure S6f, Supporting Information, *p* = 0.0035) and on the air–liquid interface (Figure 4g).

### 2.5 Soft, Fast‐Relaxing Hydrogels Result in Apical LTL Enrichment and Increased Ciliary Length and Frequency

**Figure 5:**
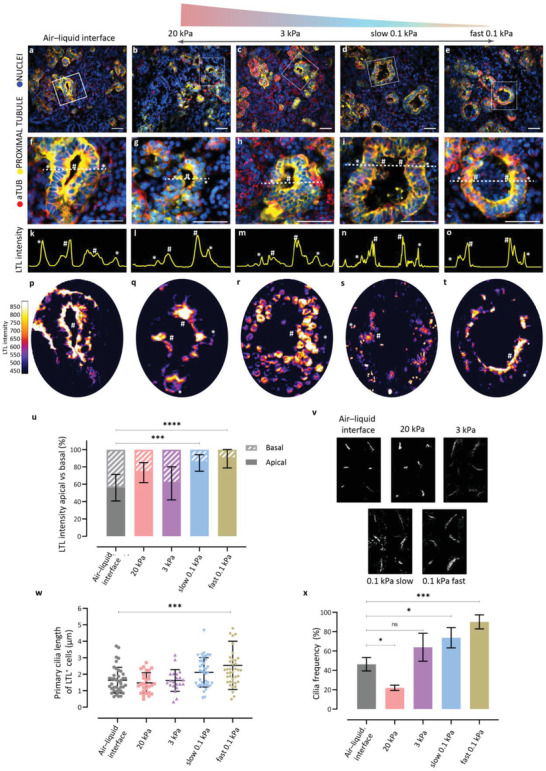
Stiffness and stress relaxation affect the polarity of the tubule lumen structures, ciliary length, and frequency. Organoids encapsulated until day 7+18 in 0.1 kPa hydrogels (d,i,n,s and e,j,o,t) showed apical enrichment of LTL^+^ tubules compared to organoids grown in the other hydrogels. The organoids on the air–liquid interface showed a clear apical and basal orientated LTL^+^ staining (a.f,k,p). When comparing apical versus basal LTL intensity, a significantly increased expression (*** = 0.0002 and **** < 0.0001 for slow‐ and fast‐relaxing hydrogel respectively; one‐way ANOVA) is observed in the soft hydrogels. Intensity plots (k–o) show the LTL intensity distribution over the dotted lines. The heat maps show overall LTL staining distribution over a complete tubule structure. DAPI staining (blue) for nuclei. The white box denotes the area of interest enlarged in the respective right panel. Scale bars: 50 µm. Representative images of *N* = 3 organoid batches with n = 3 organoids per batch. See single channels in Figure S9, Supporting Information. v) Primary cilia are observed to vary in length in the different hydrogels compared to the air–liquid interface. Representative images of *N* = 3 organoid batches (Figure S13, Supporting Information) with *n* = 3 organoids per batch. w) When measuring cilia length for cells with positive LTL staining, significantly longer primary cilia is observed for the 0.1 kPa fast‐relaxing hydrogel (*** < 0.0005; one‐way ANOVA), compared to the air–liquid interface. *N* = 3 organoid batches per condition, 5 images per batch, 40 cilia of LTL^+^ cells are measured per condition. x) Cilia frequency is altered in the different hydrogels compared to the air–liquid interface. Cilia deficiency is observed in the 20 kPa hydrogel, while a significant increase in cilia is observed in the soft, slow‐ and fast‐relaxing hydrogels. *N* = 3 organoid batches per condition, 3 images per condition, all cell counted (ns = non‐significant, * < 0.05, *** < 0.001, one‐way ANOVA).

We then found that the lumen structure varied in organoids cultured on the different hydrogels, which we attributed to the stiffness and stress relaxation characteristics of the hydrogels. A significant increase of apical enrichment was observed in the LTL^+^ lumen of the organoids cultured in the two soft hydrogels (**Figure** **5**f–k–p–u,v and Figure S10g–j, Supporting Information), while no clear lumina were observed in the stiffest hydrogel (20 kPa, Figure 5b–h–m–r and Figure S10c,d, Supporting Information). This indicated a link between stiffness and stress relaxation on the lumen maturation, in which the softer, fast‐relaxing hydrogels have an instructive function on the differentiation and polarization.

We hypothesized a link between the observed lumen structures and the accumulated stress, which the organoids experience through confinement in the stiffer hydrogels. Primary cilia play an essential role in sensing environmental cues (e.g., mechanotransduction), planar cell polarity of epithelial cells,^[^ ^31^ ^]^ lumen formation, and EMT/fibrosis after acute kidney injury,^[^ ^32^ ^]^ in which many chemical and physical can modulate their length and frequency. Therefore, we stained for primary cilia (acetylated *α*‐tubulin (aTUB) and ADP‐ribosylation factor‐like GTPase 13B (ARL13B), Figure S14a–p, Supporting Information) to investigate whether ciliary frequency and length varied in the different hydrogels. High‐resolution z‐stack confocal images showed differences in ciliary length (Figure 5v and Figure S14a–p, Supporting Information). The average length was 2.3 µm in the slow‐relaxing hydrogels and 2.7 µm in the fast‐relaxing hydrogels, both of which were significantly longer than in cells on the air–liquid interface (1.6 µm; *p* = 0.0002 and *p* < 0.0001, respectively) (Figure S14q, Supporting Information, all cell types). During kidney development, ciliary length in the nephron lumen significantly increases from 0.59 to 3.04 µm in renal vesicles to fetal nephrons, respectively.^[^ ^32^, ^33^ ^]^ When only comparing the proximal tubular ciliary length, we observed a significant increase in length of 2.5 µm (*p* = 0.0002) in the soft fast‐relaxing hydrogel compared to 1.6 µm for the air–liquid interface (Figure 5w).

EMT can also trigger reduced ciliary frequency (deficiency) and length.^[^ ^34^ ^]^ In our study, encapsulation in the stiffest hydrogel (20 kPa) led to a significant deficiency of ciliary frequency (% of cilia‐containing cells, *p* = 0.029, Figure 5x), while encapsulation in the 3 kPa hydrogel had no effect as compared to the air–liquid interface. Both these hydrogels showed EMT‐related markers. In contrast, a significant increase in ciliary frequency was observed in the soft, slow‐relaxing and fast‐relaxing hydrogels (*p* = 0.015, *p* = 0.0006, respectively, Figure 5x). This clearly showed that the organoids can feel the mechanics of their confining hydrogel environment, in which a higher stiffness (20 kPa) resulted in deficient cilia frequency and length, while softer hydrogels (0.1 kPa) significantly increased cilia frequency. However, only the fast‐relaxing hydrogel significantly increased cilia length and frequency. This may be due to the fast stress relaxation of the hydrogel network, which dissipates the tension built up due to confinement as the organoid expands throughout the hydrogel, thereby effectively reducing tension in the organoids. If we combine the significantly increased ciliary length and frequency with the clear reduction of mesenchymal cells (VIM) and aSMA expression, and the apical enrichment of LTL in the organoids encapsulated in the soft fast‐relaxing hydrogel, we can postulate a link between hydrogel stiffness and stress relaxation on lumen maturation. When kidney organoids were confined in a hydrogel, the ability of the material to disperse tension through rapid stress‐relaxing resulted in a reduction of off‐target cell types and increased lumen maturation.

## 3 Outlook

The effect of hydrogel stiffness and stress relaxation has been well studied on individual cells, but fewer reports exist on the combined effects on aggregates and organoids. The potential to engineer a matrix that influences these large multicellular aggregates may have been overlooked due to the perception that relatively few cells interact with the matrix. As iterated in our findings, mimicking the dynamics of the ECM has repeatedly been shown to be important. Our studies indicate that the stiffness and stress relaxation of the surrounding environment had a direct impact on kidney organoids. Stiffer hydrogels caused an undesirable EMT and loss of lumen structures, while softer hydrogels positively reduced undesired ECM deposition, increased the maturity of lumen structures, and reduced mesenchymal cells. Overall, our results show the importance and potential of tuning the hydrogel properties to influence kidney organoids. Properly engineered matrices for organoids could lead to a platform for culturing functional engraftments and to disease modeling applications by tuning the hydrogel to mimic pathological scenarios such as fibrosis.

## 4 Experimental Section

### Oxidized Alginate (Oxi‐Alg) Synthesis

Sodium alginate was oxidized (10%) as previously described.^[^ ^20^ ^]^ Briefly, purified sodium alginate (1.0 g, 1 equiv, 5.68 × 10^−3^ mol monomer, Manugel GMB, FMC, Lot No. G940200) was dissolved in 100 mL deionized H_2_O overnight. Sodium (meta)periodate (0.121 g, 0.1 equiv, 5.68 × 10^−4^ mol, Sigma‐Aldrich) was added. The mixture was covered with aluminum foil and stirred in the dark for 17 h at room temperature (RT). The reaction was quenched by the addition of ethylene glycol (0.035 g, 0.1 equiv 5.68 × 10^−4^ mol, Sigma‐Aldrich) and stirred for 1 h in the dark at RT. The resultant product was dialyzed in a 10 kDa MWCO dialysis tube (Spectra/Por, regenerated cellulose, VWR) for 3 d in 100 mM, 50 mM, 25 mM, 12.5 mM, and 0 mM NaCl in deionized H_2_O (changed twice daily), and was flash‐frozen in liquid N_2_ and lyophilized. Oxidation was confirmed by ^1^H‐NMR in deuterated water (D_2_O, Figure S1, Supporting Information) by the appearance of the protons between 5.15 and 5.75 ppm, attributed to the formation of hemiacetal groups upon reaction of the aldehydes to neighboring hydroxyl groups. Moreover, molecular weights of the product were determined via GPC (Figure S2, Supporting Information).

### ^1^H‐NMR Analysis

^1^H‐NMR spectra were recorded on a Bruker Avance III HD 700‐MHz spectrometer equipped with a cryogenically cooled three‐channel TCI probe in D_2_O with sodium trimethylsilylpropanesulfonate‐d6 as an internal standard (DSS‐d6, 2 × 10^–3^ M). Water suppression pulse sequence was applied to spectra. Spectra analyses were performed with MestReNova 11.0 software. Chemical shifts were reported in parts per million (ppm) relative to DSS‐d6 (CH_2_, 0 ppm).

### GPC Analysis

Agilent PEG calibration kit (PEG molecular weights up to 300 000 MW, Agilent Technologies) was used for calibration. The samples were dissolved at a concentration of 2 mg·mL^−1^ in 0.1 M NaNO_3_ H_2_O and filtered through 0.45 µm filters to remove any unwanted impurities. Samples MW were measured in aqueous 0.1 M NaNO_3_ eluent with a flow rate of 0.5 mL·min^−1^ at RT on a Prominence‐I LC‐2030C3D LC (Shimadzu Europa GmbH) and Shodex SB‐803/SB‐804 HQ columns (Showax Denko America, Inc). LabSolutions GPC software (Shimadzu Europa GmbH) was used to calculate the molecular weight and dispersity values (Table S1, Supporting Information).

### Rheometry

Rheological characterization on the hydrogels was performed on a DHR2 rheometer from TA Instruments. Time sweeps of preformed hydrogels were taken over 360 s with an 8 mm parallel plate geometry at 20 °C with an applied strain of 1% at 3.14 rad s^−1^. During loading, the gap size was adjusted to achieve 0.1 N of normal force and varied between samples from 800 to 1150 µm with a mean of 1004 ± 76 µm. The shear storage modulus (G′) for a given sample was taken to be the mean recorded value with a minimum of three sample replicates per formulation (Figures 1c and 5b). Stress relaxation measurements were performed using a 20 mm cone‐plate geometry equipped with solvent trap. Precursor solutions were mixed as described in Table S2, Supporting Information, to obtain the desired final concentrations of oxidized alginate and cross‐linker. Following the final addition of the cross‐linker, samples were vortexed for 10 s before immediately loading 80 µL into the rheometer. Time sweeps were measured over 3.5–9 h (maintained at 20 °C) to monitor crosslinking progress with an applied strain of 1% at 10 rad s^−1^. Once a plateau was reached, a frequency sweep was performed from 100–0.1 rad s^−1^ with an applied strain of 1% and 10 pts dec^−1^ (Figure S3c, Supporting Information). Finally, to measure the stress relaxation behavior, the relaxation modulus was monitored over 15.5 h with an initially applied strain of 20% maintained over the course of the measurement (Figures 1d and 5c). Statistical analyses were performed in GraphPad 8.2.0 using one‐way ANOVA.

### Swelling Test of Hydrogels under Culture Conditions

A swelling test to investigate real‐world swelling under culture conditions was performed on transwell filters, as previously described.^[^ ^9^ ^]^ Briefly, hydrogel solutions were prepared (Table S1, Supporting Information) and 500 µL of this solution was added onto the transwells with a 0.4 µm pore size (Corning, 12‐well culture plate) without organoids and left to cross‐link for 1 h. STEMdiff APEL2 medium (1% (v/v) PFHM‐II protein‐free hybridoma medium (Thermo Fisher Scientific) and 1% (v/v) antibiotic/antimycotic (Thermo Fisher Scientific) was added below the transwells with hydrogels and incubated up to 98 h (37 °C). Transwells with hydrogels were weighed at 1, 2, 4, 6, 24, 48, 72, and 96 h. The hydrogel swelling ratio in % (*S*_r_) over time was calculated by: $S_{r}=\frac{w_{x}-w_{o}}{w_{o}}$, in which *w*_0_ = initial hydrogel weight and *w*_x_ = hydrogel weight at the *x* time point (Figures S3d and S8d, Supporting Information). GraphPad 8.2.0 software was used for statistical analyses using two‐way ANOVA or unpaired *t*‐test.

### Cell Culture

As described previously,^[^ ^9^ ^]^ the hiPSC line LUMC0072iCTRL01 was generated from fibroblasts using the Simplicon RNA reprogramming kit (Millipore) by the hiPSC core facility at the Leiden University Medical Center (the Netherlands). The cells were expanded in E8 medium (Thermo Fisher Scientific) on vitronectin‐coated (0.5 µg·cm^−2^) plates and passaged with TrypLE Express (Thermo Fisher Scientific) twice weekly. For 24 h after each passage, cells were cultured in E8 medium supplemented with RevitaCell Supplement (Thermo Fisher Scientific). Subsequently, cells were cultured in E8 medium refreshed daily.

### Differentiation and Organoid Formation

Kidney organoids were produced from hiPSCs according to an established protocol (Figure 1a).^[^ ^15b^ ^]^ Briefly, hiPSCs were seeded on 6‐well plates with vitronectin‐coating (0.5 µg·cm^−2^), at 7300 cells per cm^2^ in E8 medium supplemented with RevitaCell Supplement. Differentiation was started after 24 h (day 0) by changing to STEMdiff APEL2 medium (STEMCELL Technologies) supplemented with CHIR99021 (8 µm, r&D Systems), 1% (*v*/*v*) antibiotic/antimycotic (Thermo Fisher Scientific), and 1% (*v*/*v*) PFHM‐II protein‐free hybridoma medium (Thermo Fisher Scientific). On day 4 of differentiation, the medium was changed to STEMdiff APEL medium supplemented with heparin (1 µg mL^−1^, Sigma‐Aldrich) and FGF‐9 (200 ng·mL^−1^, R&D Systems). By day 7, the cells formed a confluent monolayer and were subjected to a 1 h pulse of CHIR99021 (5 µm) in STEMdiff APEL medium before being harvested by trypsinisation. An aggregate of 500 000 cells was transferred to a Transwell filter with 0.4 µm pore size (Corning) in a 12‐well culture plate to form the kidney organoids. Kidney organoids were cultured for 4 d (termed day 7+4) on an air–liquid interface with STEMdiff APEL2 medium supplemented with FGF‐9 and heparin in the bottom compartment (450 µL) with media changes every 2 d. At day 7+5, STEMdiff APEL2 medium with no supplemented growth factors was used when media changes were performed every 2 d. The organoids were maintained until day 7+18, with or without hydrogel encapsulation from day 7+14 (Figure 1a,b).

### Hydrogel Encapsulation

Oxidized alginate (120 mg, 10% oxidation, 6 wt% stock solution, UV sterilized, Oxi‐alg) was dissolved in 2 mL STEMdiff APEL2 medium and left to dissolve overnight. O,O′‐1,3‐propanediylbishydroxylamine dihydrochloride (oxime, Sigma‐Aldrich) or adipic dihydrazide (hydrazone, Sigma‐Aldrich) stock solutions of 8 × 10^–2^ mol·L^−1^ were prepared in STEMdiff APEL2 medium (Table S2, Supporting Information) and passed through a 0.2 µm sterilization filter. The oxi‐alg, crosslinker stock solutions, and STEMdiff APEL2 medium were added to obtain the desired hydrogel systems (Table S2) and vortexed before organoid encapsulation to form a 2% or 4% (*w*/*v*) sodium alginate solution. The hydrogel solutions were pipetted over the organoids on the top of the Transwell membrane at day 7+14 of culture (500 µL each). STEMdiff APEL2 medium (450 µL) was added below the Transwell filters, and organoids encapsulated in the hydrogels were cultured for 4 additional days (until day 7+18, Figure 1A,B).

### Cryo‐Sectioning Recovered Organoids

At day 7+18, the hydrogels were removed and 4% paraformaldehyde (PFA) was added above and below the Transwell with the recovered organoid for 20 min at 4 °C. Organoids were cryo‐sectioned as described previously.^[^ ^9^ ^]^ Briefly, organoids were dehydrated overnight (PBS containing 15% (*w*/*v*) sucrose) at 4 °C followed by a second 2 d dehydration incubation (30% (*w*/*v*) sucrose). The dehydrated organoids were embedded in freezing solution (15% (*w*/*v*) sucrose and 7.5% (*w*/*v*) gelatin in PBS). The embedded organoids were placed in a beaker with isopentane and left to freeze in liquid N_2_ for several minutes. The frozen organoids were horizontally sectioned to 20 µm thickness at −18 °C.

### Immunohistochemistry

The embedding solution of the frozen organoid sections was removed by incubating for 15–20 min in PBS at 37 °C. The sections were washed (PBS), permeabilised (PBS with 0.5% (*v*/*v*) IGEPAL) for 15 min (RT), blocked (PBS with 5% (*w*/*v*) donkey serum, 1% BSA and 0.3 M glycine) for 20 min (RT), and incubated overnight at 4 °C (in the dark) with primary antibodies (PBS with 1% BSA and 0.3 M glycine) against: nephrin (NPHS1), lotus tetragonolobus lectin (LTL), Meis Homeobox 1/2/3 (MEIS1/2/3), E‐cadherin (ECAD), solution carrier family 12 Member 1 (SLC12A1), type 1a1 and type 6a1 collagen, aSMA, vimentin, acetylated tubulin (a‐tubulin), ADP‐ribosylation factor‐like GTPase 13B (ARL13B), and Zinc finger protein SNAI1 (**Table** **1**). Subsequently, the slides were washed three times with PBS (1% BSA and 0.3 M glycine) and incubated with secondary antibodies including DAPI (0.1 µg mL^−1^) for 1 h at RT in the dark: Alexa Fluor 488 (Thermo Fisher Scientific, 1:300, sheep/mouse/rabbit), Alexa Fluor 568 (Thermo Fisher Scientific, 1:300, mouse/rabbit), and Streptavidin Alexa Fluor 647 (Thermo Fisher Scientific, 1:100). Slides were washed three times in PBS and mounted with Mowiol mounting medium. Images were taken with an automated Nikon Eclipse Ti2‐E microscope (20× or 40× air objective) or light microscope Leica TCS SP8 STED (100× objective).

**Table 1:** Primary antibodies used in this study

Primary Antigen	Mono‐ or polyclonal	Source (catalog no.)	Species	Identifies	Dilution
Acetylated *α*‐Tubulin (aTUB)	Mono	MERCK (T7451)	Mouse	Primary cilia	(1:750)
ARL13B	Poly	Proteintech (17711‐1‐AP)	Rabbit	Primary cilia	(1:400)
aSMA	Mono	Sigma‐Aldrich (A2547)	Mouse	Fibroblasts	(1:300)
ECAD	Mono	BD Biosciences (610181)	Mouse	Distal tubule	(1:300)
LTL	‐	Vectorlabs (B‐1325)	Biotin conjugate	Proximal tubule	(1:300)
MEIS1/2/3	Mono	Santa‐Cruz Biotechnology (SC‐101850)	Mouse	Interstitial cells	(1:300)
NPHS1	Poly	R&D systems (AF426)9	Sheep	Podocytes	(1:300)
SLC12A1	Poly	Thermo fisher scientific (HPA014967)	Rabbit	Loop of henle	(1:200)
SNAIL	Mono	Thermo fisher scientific (PA5‐85493)	Rabbit	EMT transcription factor	(1:300)
Type I Collagen (COL1A1)	Mono	Abcam (ab6308)	Mouse	Type 1 collagen	(1:300)
Type VI Collagen (COL6A1)	Mono	Genetex (GTX109963)	Rabbit	Type 6 collagen	(1:300)
Vimentin (VIM)	Mono	Thermo fisher scientific (MA3745)	Mouse	EMT ECM marker	(1:300)

### Immunohistochemistry Intensity Measurements

Immunohistochemistry images were processed in ImageJ. Single channels were set to scale when converting to 16‐bit images. Thresholds were set (Table S3, Supporting Information) and mean grey intensities were measured per antibody. Statistical analyses were performed in GraphPad 8.2.0 using two‐way ANOVA.

### Cell Viability Assay

EthD1/calcein AM staining was used to determine cell viability. The encapsulating hydrogel and medium were removed. Recovered organoids on Transwells were incubated in EthD1 (4 µM) and calcein AM (2 µM) in PBS solution (top and bottom of the Transwell) for 30 min at RT. Organoids were imaged in PBS with an automated Nikon Eclipse Ti2‐E microscope at 4×, 20×, or 40× air objective.

### Analysis of Emt‐Related Gene Expression in Kidney Organoids

Single‐cell RNAseq data of iPSC‐derived kidney organoids generated using the Takasato protocol^[^ ^18^ ^]^ were downloaded from the Gene Expression Omnibus (GEO: GSE118184) and analyzed as previously described.^[^ ^9^ ^]^ Briefly, transcript count tables were analyzed for each time point (day 0, 7, 7+5, 7+12, 7+19, and 7+27) by R software (3.6.2) and the Seurat package (version 3.2.0); with the exclusion of low‐quality cells. The gene expression matrices were log‐transformed using a scaling factor of 10000 and normalized for sequencing depth per cell. Subsequently, the highest cell‐to‐cell variations were identified, scaled, and centered. These data were used for principal component analysis. Non‐linear dimensional reduction was performed on selected principal components representing the true dimensionality of each dataset. Normalized markers of gene expression of interest was presented in tSNE space.

### LTL Polarity of Lumen Structures

Immunohistochemistry images were processed in ImageJ. Plot profiles were analyzed in ImageJ and heatmaps were generated by the interactive 3D surface plot plugin. Percentage of basal versus apical intensity was calculated by deducting plot profile from apical side from the full plot profile per lumen structure. Statistical analyses were performed in GraphPad 8.2.0 using two‐way ANOVA.

### Primary Cilia Length and Frequency Measurements

Z‐stack confocal images measured (100× objective) were processed in ImageJ to Z projection. Length per cilia was measured using the straight‐line function Cilia frequency was measured using the cell counter plugin by measuring the percentage of nuclei with primary cilia compared to the overall nuclei. Statistical analyses were performed in GraphPad 8.2.0 using two‐way ANOVA.

### Statistical Analysis

All statistical analysis were performed in GraphPad 8.2.0 using one or two‐way ANOVA. See specifics per analysis in the separate materials and methods section and figure legends.

## Conflict of Interest

F.A.A.R, F.L.C.M., V.L.S.L. and M.B.B. are co‐inventors on a patent submission based upon these findings.

## Supporting information

Supporting Information

## Data Availability

The data that support the findings of this study are openly available in DataVerse at https://doi.org/10.34894/0D0JXE, reference number [35].

## References

[1] a) V. Magno , A. Meinhardt , C. Werner , Adv. Funct. Mater. 2020, 30, 2000097;

[2] M. M. Capeling , M. Czerwinski , S. Huang , Y.‐H. Tsai , A. Wu , M. S. Nagy , B. Juliar , N. Sundaram , Y. Song , W. M. Han , S. Takayama , E. Alsberg , A. J. Garcia , M. Helmrath , A. J. Putnam , J. R. Spence , Stem Cell Rep. 2019, 12, 381. 10.1016/j.stemcr.2018.12.001 PMC6373433 30612954

[3] a) N. Glorevski , N. Sachs , A. Manfrin , S. Giger , M. E. Bragina , P. Ordonez‐Moran , H. Clevers , M. P. Lutolf , Nature 2016, 539, 560; 27851739 10.1038/nature20168

[4] N. Broguiere , L. Isenmann , C. Hirt , T. Ringel , S. Placzek , E. Cavalli , F. Ringnalda , L. Villiger , R. Züllig , R. Lehmann , G. Rogler , M. H. Heim , J. Schüler , M. Zenobi‐Wong , G. Schwank , Adv. Mater. 2018, 30, 1801621. 10.1002/adma.201801621 30203567

[5] R. Cruz‐Acuna , M. Quiros , A. E. Farkas , P. H. Dedhia , S. Huang , D. Siuda , V. Garcia‐Hernandez , A. J. Miller , J. R. Spence , A. Nusrat , A. J. Garcia , Nat. Cell Biol. 2017, 19, 1326. 29058719 10.1038/ncb3632 PMC5664213

[6] J. Candiello , T. S. P. Grandhi , S. K. Goh , V. Vaidya , M. Lemmon‐Kishi , K. R. Eliato , R. Ros , P. N. Kumta , K. Rege , I. Banerjee , Biomaterials 2018, 177, 27. 29883914 10.1016/j.biomaterials.2018.05.031

[7] A. Ranga , M. Girgin , A. Meinhardt , D. Eberle , M. Caiazzo , E. M. Tanaka , M. P. Lutolf , Proc. Natl. Acad. Sci. U. S. A. 2016, 113, E6831. 27742791 10.1073/pnas.1603529113 PMC5098636

[8] L. Broutier , A. Andersson‐Rolf , C. J. Hindley , S. F. Boj , H. Clevers , B.‐K. Koo , M. Huch , Nat. Protoc. 2016, 11, 1724. 27560176 10.1038/nprot.2016.097

[9] T. Geuens , F. A. A. Ruiter , A. Schumacher , F. L. C. Morgan , T. Rademakers , L. E. Wiersma , C. W. van den Berg , T. J. Rabelink , M. B. Baker , V. L. S. LaPointe , Biomaterials 2021, 275, 120976. 34198162 10.1016/j.biomaterials.2021.120976

[10] J. A. Burdick , W. L. Murphy , Nat. Commun. 2012, 3, 1269. 23232399 10.1038/ncomms2271

[11] a) H. W. Ooi , S. Hafeez , C. A. van Blitterswijk , L. Moroni , M. B. Baker , Mater. Horiz. 2017, 4, 1020;

[12] D. D. McKinnon , D. W. Domaille , T. E. Brown , K. A. Kyburz , E. Kiyotake , J. N. Cha , K. S. Anseth , Soft Matter 2014, 10, 9230. 25265090 10.1039/c4sm01365d PMC4445372

[13] J. Lou , R. Stowers , S. Nam , Y. Xia , O. Chaudhuri , Biomaterials 2018, 154, 213. 29132046 10.1016/j.biomaterials.2017.11.004

[14] H. P. Lee , L. Gu , D. J. Mooney , M. E. Levenston , O. Chaudhuri , Nat. Mater. 2017, 16, 1243. 28967913 10.1038/nmat4993 PMC5701824

[15] a) M. Takasato , P. X. Er , H. S. Chiu , B. Maier , G. J. Baillie , C. Ferguson , R. G. Parton , E. J. Wolvetang , M. S. Roost , S. M. C. de Sousa Lopes , M. H. Little , Nature 2015, 526, 564; 26444236 10.1038/nature15695

[16] a) M. H. Little , P. Kairath , Kidney Int. 2016, 90, 289; 27234568 10.1016/j.kint.2016.03.030

[17] N. R. Hill , S. T. Fatoba , J. L. Oke , J. A. Hirst , C. A. O'Callaghan , D. S. Lasserson , F. D. R. Hobbs , PLoS One 2016, 11, e0158765. 27383068 10.1371/journal.pone.0158765 PMC4934905

[18] H. Wu , K. Uchimura , E. L. Donnelly , Y. Kirita , S. A. Morris , B. D. Humphreys , Cell Stem Cell 2018, 23, 869. 30449713 10.1016/j.stem.2018.10.010 PMC6324730

[19] M. Takasato , P. X. Er , H. S. Chiu , M. H. Little , Nat. Protoc. 2016, 11, 1681. 27560173 10.1038/nprot.2016.098 PMC5113819

[20] S. Hafeez , H. Ooi , F. Morgan , C. Mota , M. Dettin , C. van Blitterswijk , L. Moroni , M. Baker , Gels 2018, 4, 85. 10.3390/gels4040085 PMC6318581 30674861

[21] M. H. Hettiaratchi , A. Schudel , T. Rouse , A. J. García , S. N. Thomas , R. E. Guldberg , T. C. McDevitt , APL Bioeng. 2018, 2, 026110. 31069307 10.1063/1.4999925 PMC6324205

[22] J. Sun , H. Tan , Materials 2013, 6, 1285. 28809210 10.3390/ma6041285 PMC5452316

[23] a) L. Sheng , S. Zhuang , Front. Physiol. 2020, 11, 569322. 33041867 10.3389/fphys.2020.569322 PMC7522479

[24] a) M. Bozic , M. Caus , R. R. Rodrigues‐Diez , N. Pedraza , M. Ruiz‐Ortega , E. Garí , P. Gallel , M. J. Panadés , A. Martinez , E. Fernández , J. M. Valdivielso , Nat. Commun. 2020, 11, 1943; 32327648 10.1038/s41467-020-15732-9 PMC7181766

[25] F. A. Y. Yengej , J. Jansen , M. B. Rookmaaker , M. C. Verhaar , H. Clevers , Cells 2020, 9, 1326. 10.3390/cells9061326 PMC7349753 32466429

[26] M. G. Ondeck , A. Kumar , J. K. Placone , C. M. Plunkett , B. F. Matte , K. C. Wong , L. Fattet , J. Yang , A. J. Engler , Proc. Natl. Acad. Sci. U. S. A. 2019, 116, 3502. 30755531 10.1073/pnas.1814204116 PMC6397509

[27] K. Ahmed , H. Dehghani , P. Rugg‐Gunn , E. Fussner , J. Rossant , D. P. Bazett‐Jones , PLoS One 2010, 5, e10531. 20479880 10.1371/journal.pone.0010531 PMC2866533

[28] W.‐C. Chen , H.‐H. Lin , M.‐J. Tang , Am. J. Physiol. Renal Physiol. 2014, 307, F695. 25056346 10.1152/ajprenal.00684.2013

[29] M. K. Ma , H. K. Law , K. S. Tse , K. W. Chan , G. C. Chan , D. Y. Yap , M. M. Mok , L. P. Kwan , S. C. Tang , B. Y. Choy , T. M. Chan , Int. J. Urol. 2018, 25, 450. 29444550 10.1111/iju.13536

[30] a) O. Chaudhuri , L. Gu , D. Klumpers , M. Darnell , S. A. Bencherif , J. C. Weaver , N. Huebsch , H.‐p. Lee , E. Lippens , G. N. Duda , D. J. Mooney , Nat. Mater. 2016, 15, 326; 26618884 10.1038/nmat4489 PMC4767627

[31] S. Wang , Z. Dong , Am. J. Physiol. Renal Physiol. 2013, 305, F1085. 23904226 10.1152/ajprenal.00399.2013 PMC3798724

[32] a) S. J. Han , J. H. Kim , J. I. Kim , K. M. Park , Sci. Rep. 2016, 6, 27775; 27270990 10.1038/srep27775 PMC4897697

[33] A. N. Marra , Y. Li , R. A. Wingert , Genesis 2016, 54, 457. 27389733 10.1002/dvg.22957 PMC5053263

[34] S. J. Han , J. K. Jung , S. S. Im , S. R. Lee , B. C. Jang , K. M. Park , J. I. Kim , Biochem. Biophys. Res. Commun. 2018, 496, 450. 29337054 10.1016/j.bbrc.2018.01.079

[35] F. Ruiter , V. LaPointe , M. Baker , 2022, 10.34894/0D0JXE.

