# Supplementary material for: Soft, Dynamic Hydrogel Confinement Improves Kidney Organoid Lumen Morphology and Reduces Epithelial–Mesenchymal Transition in Culture
Source: Adv Sci (Weinh). 2022 May 14;9(20):2200543. doi: 10.1002/advs.202200543 (PMC9284132; doi:10.1002/advs.202200543)
Supplement: Supplementary file 1 — Supporting Information [file ADVS-9-2200543-s001.pdf]

ADVANCED SCIENCE
Open Access

Supporting Information

for *Adv. Sci.*, DOI 10.1002/advs.202200543

Soft, Dynamic Hydrogel Confinement Improves Kidney Organoid Lumen Morphology and Reduces Epithelial–Mesenchymal Transition in Culture

Floor A. A. Ruiter, Francis L. C. Morgan, Nadia Roumans, Anika Schumacher, Gisela G. Slaats, Lorenzo Moroni, Vanessa L. S. LaPointe and Matthew B. Baker**

Additional information

Extra information oxidisation confirmation.

The sugar monomers' hydroxyl groups at C-2 and C-3 in the alginate chain were oxidised resulting in the breakage of the C–C bonds to form two aldehyde groups, which rapidly formed hemiacetals with neighbouring alcohol groups. These hemiacetal groups were confirmed via the appearance of the proton peaks at 5.2–5.8 ppm in the ^{1}H-NMR spectra (**Figure S1**), confirming the oxidation. The molecular weight (MW) decreased from approximately 320 kDa in the starting material to 70 kDa due to the oxidisation breaking part of the backbone of the alginate structure (**Figure S2 and Table S1**).

Extra information Figure 3A. Reanalysis of single-cell RNA sequencing

The data presented in Figure 3A are taken from a study performed by Humprhreys lab[18]. Here we reanalysed their single-cell RNA sequencing of organoids generated by the Takasato protocol from iPSC for relevant EMT-related (TWIST1); mesenchymal markers (VIM, ACTA2, SNAI1, and CDH2) and epithelial marker (CDH1) to investigate if EMT occurs at later stage of the organoids culture. When EMT occurs, the expression of TWIST1 and mesenchymal markers increase, while the epithelial marker E-cadherin (CDH1) decreases. Indeed, we found an increase in the percentages of cells that were positive for TWIST1, VIM, ACTA2, SNAI1, and CDH2 and a decrease in $CDH1^+$ at day 7+27 in culture.

Supplementary figures and tables

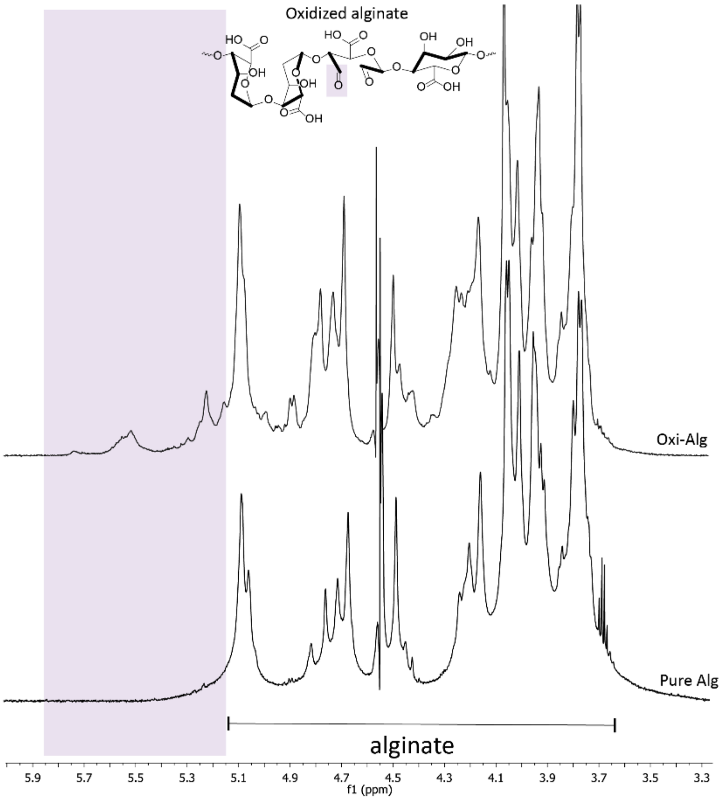

Figure S1. ^{1}H-NMR spectra of pure alginate (pure Alg; bottom) and oxidised alginate (oxi-alg; top). Oxidation of the alginate was confirmed by the appearance of the protons between 5.15–5.75 ppm, attributed to the formation of hemiacetal groups upon reaction of the aldehydes to neighbouring hydroxyl groups (top spectra, in purple area), compared to the pure alginate NMR spectra, in D_2O. DSS-d6 was used as internal standard and samples were measured at 325 K.

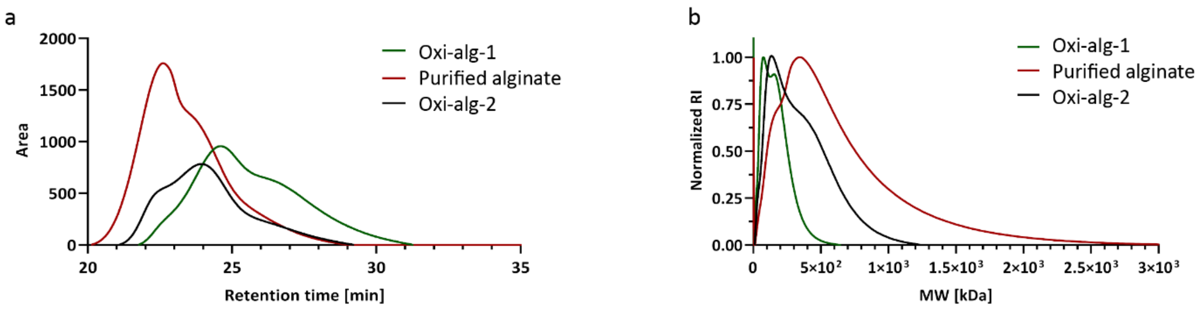

Figure S2. Reduced molecular weight confirms oxidation of the alginate. a) GPC retention time of the two oxidised alginate batches (oxi-alg-1 for the stiffness series and oxi-alg-2 for the fast-relaxing, 0.1 kPa hydrogel) compared to the pure alginate. b) Molecular weight of the oxidised alginates batches (oxi-alg-1 for the stiffness series and oxi-alg-2 for the fast-relaxing, 0.1 kPa hydrogel) compared to the pure alginate. As expected, the oxidation of the alginate backbone resulted in a reduction of the molecular weight of the resultant product; see values in Table S1.

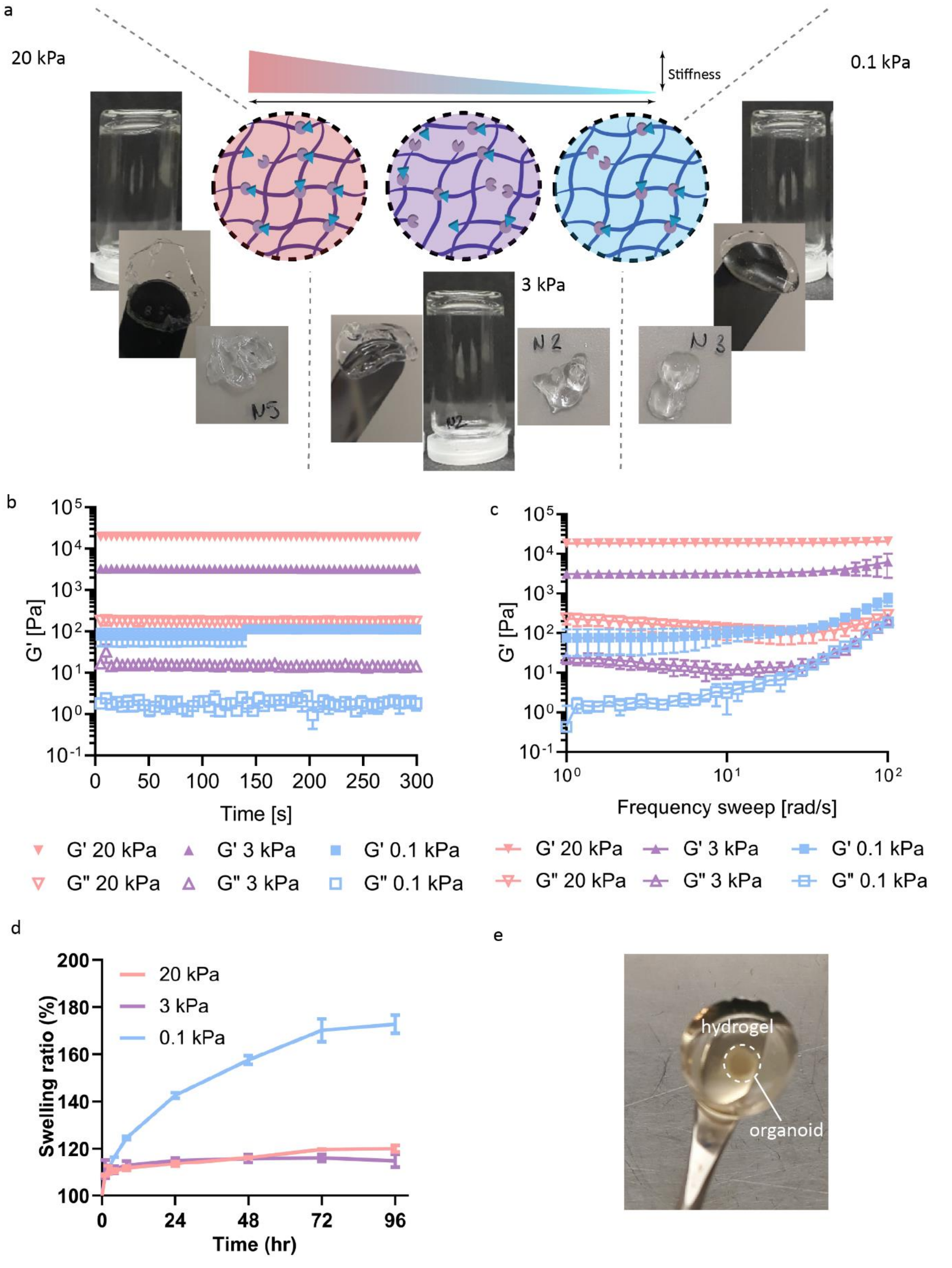

Figure S3. Hydrogel formation of different stiffnesses were confirmed and properties characterised. a) Hydrogel formation confirmed by inverted vial test (left image of each set);

formed hydrogel on spatula (centre image of each set); and removed hydrogel from vial (right image of each set). The different systems were (left to right): 4% alginate–20.2 μM oxime (pink); 2% alginate–10.1 μM oxime (purple); 2% alginate–2.02 μM oxime (blue). b) Time sweep data of the three hydrogels was used to determine the stiffness values of the hydrogels (N=2, Figure 1C). Colour coding of hydrogels as indicated in panel a. c) Frequency sweep data of the hydrogels (N=2) showed they were frequency independent. Colour coding of hydrogels as indicated in panel a. d) Swelling test showed a significant swelling of 172% for the 0.1 kPa hydrogel (two-way ANOVA, $p < 0.0001$, N=3) after 96 h incubation, compared to 120% swelling for the 3 kPa and 20 kPa hydrogels. Points and bars indicate mean ± standard error from three individual samples. e) Representative image of recovered organoid in the 20 kPa hydrogel.

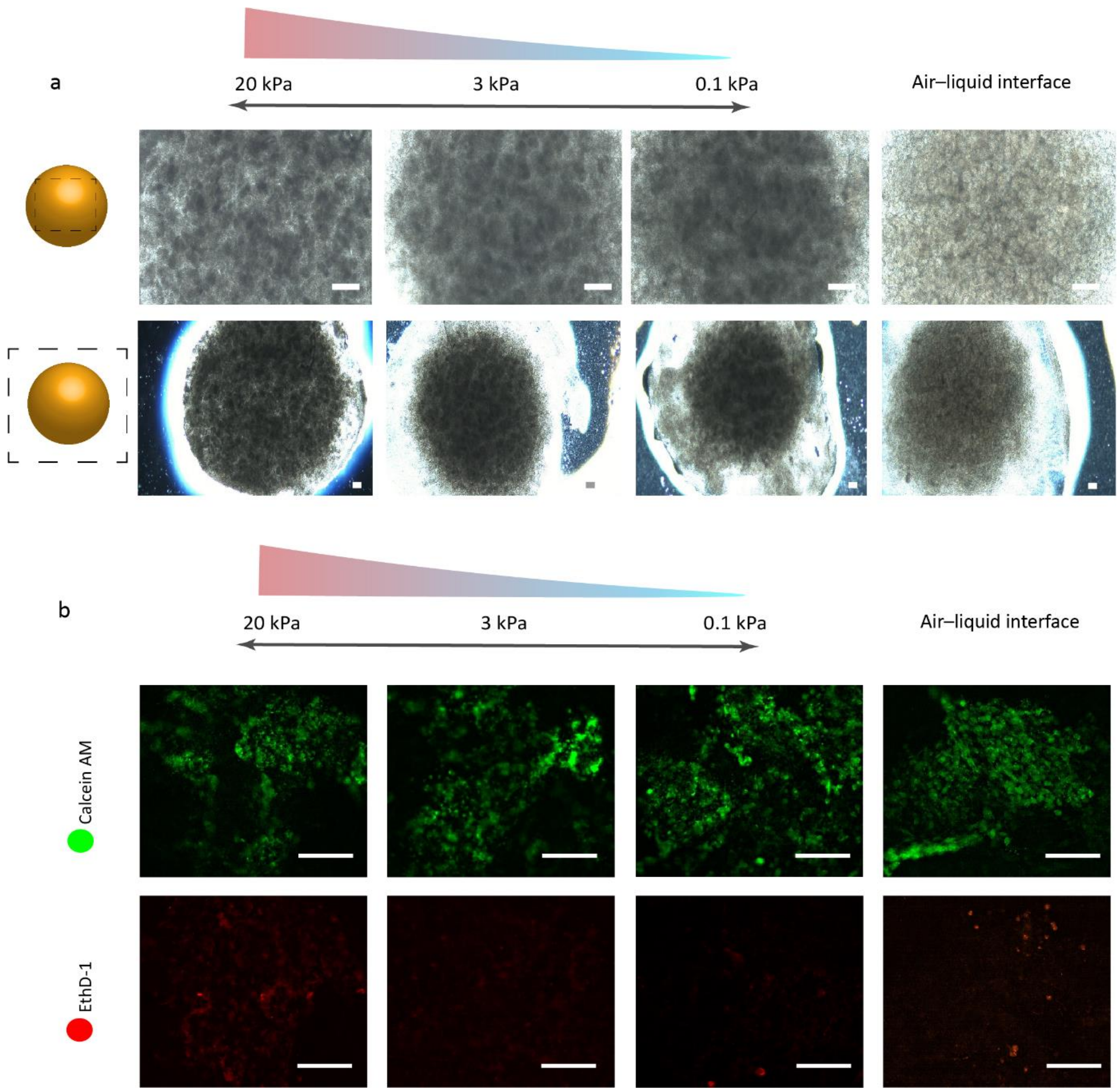

Figure S4. **Organoid morphology and viability were maintained in the encapsulated hydrogels.** a) Brightfield images of the organoids encapsulated in the different stiffness hydrogels or cultured on the air–liquid interface after 7+18 d. Schematic at the left indicates where images were taken. b) Live/dead assay with calcein AM (live) and EthD-1 (red) showed similar staining between the different culture environments. Scale bars: 100 µm. Representative images of N=3 organoid batches with n=3 organoids per batch.

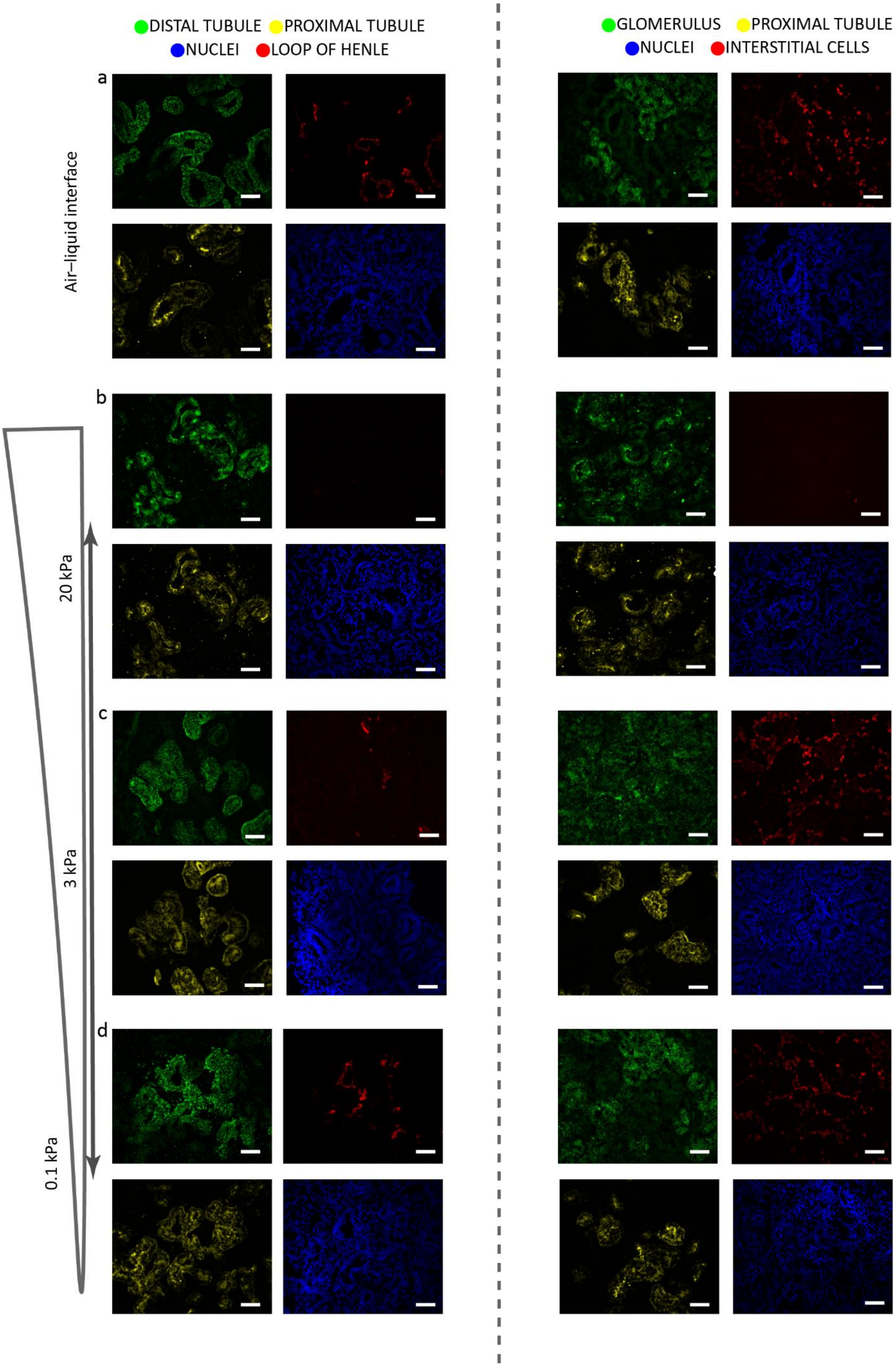

DISTAL TUBULE
PROXIMAL TUBULE
NUCLEI
LOOP OF HENLE
GLOMERULUS
PROXIMAL TUBULE
NUCLEI
INTERSTITIAL CELLS
a
Air–liquid interface
b
20 kPa
c
3 kPa
d
0.1 kPa

Figure S5. Single channels of immunohistochemistry in **Figure 2a-d** of glomeruli (nephrin: NPHS1, in green in third column from the left), proximal tubules (lotus tetragonolobus lectin: LTL, in yellow), loop of Henle (NKCC2 and SLC12A1, in red in second column from the left), distal tubules (E-cadherin: ECAD, in green in the far-left column), and interstitial cells (homeobox protein Meis 1/2/3: MEIS1/2/3, in red in the far-right column). DAPI staining (blue) for nuclei. Scale bars: 50 µm. Representative images of N=3 organoid batches with n=3 organoids per batch.

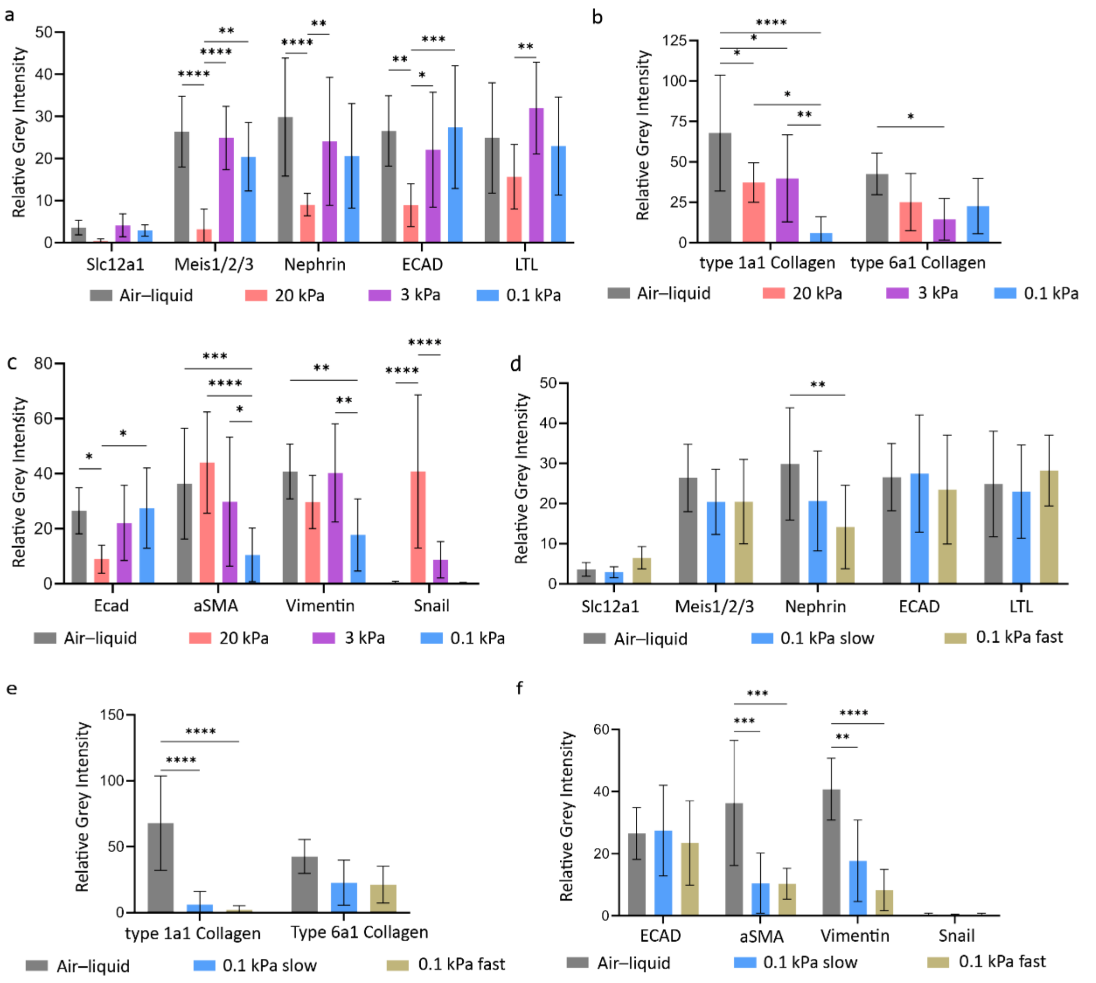

Figure S6. Relative grey intensities from immunostaining images of a/d) the different cell types glomeruli (nephrin: NPHS1), proximal tubules (lotus tetragonolobus lectin: LTL), loop of Henle (SLC12A1), distal tubules (E-cadherin: ECAD), and interstitial cells (homeobox protein Meis 1/2/3: MEIS1/2/3); b/e) expression of ECM proteins type 1a1 and type 6a1 collagen and c/f) EMT-related expression in a-c) the organoids cultured in the 0.1, 3, 20 kPa hydrogel and on the air–liquid interface or in the organoids cultured in d-f) the 0.1 kPa slow- and fast-relaxing hydrogels and on the air-liquid interface. Error bars = Standard deviation. Two-way ANOVA, *<0.05, **<0.005, ***<0.0005 and ****<0.0001. Images used of N=3 organoid batches with n=3 organoids per batch, 9 grey intensities measured per condition.

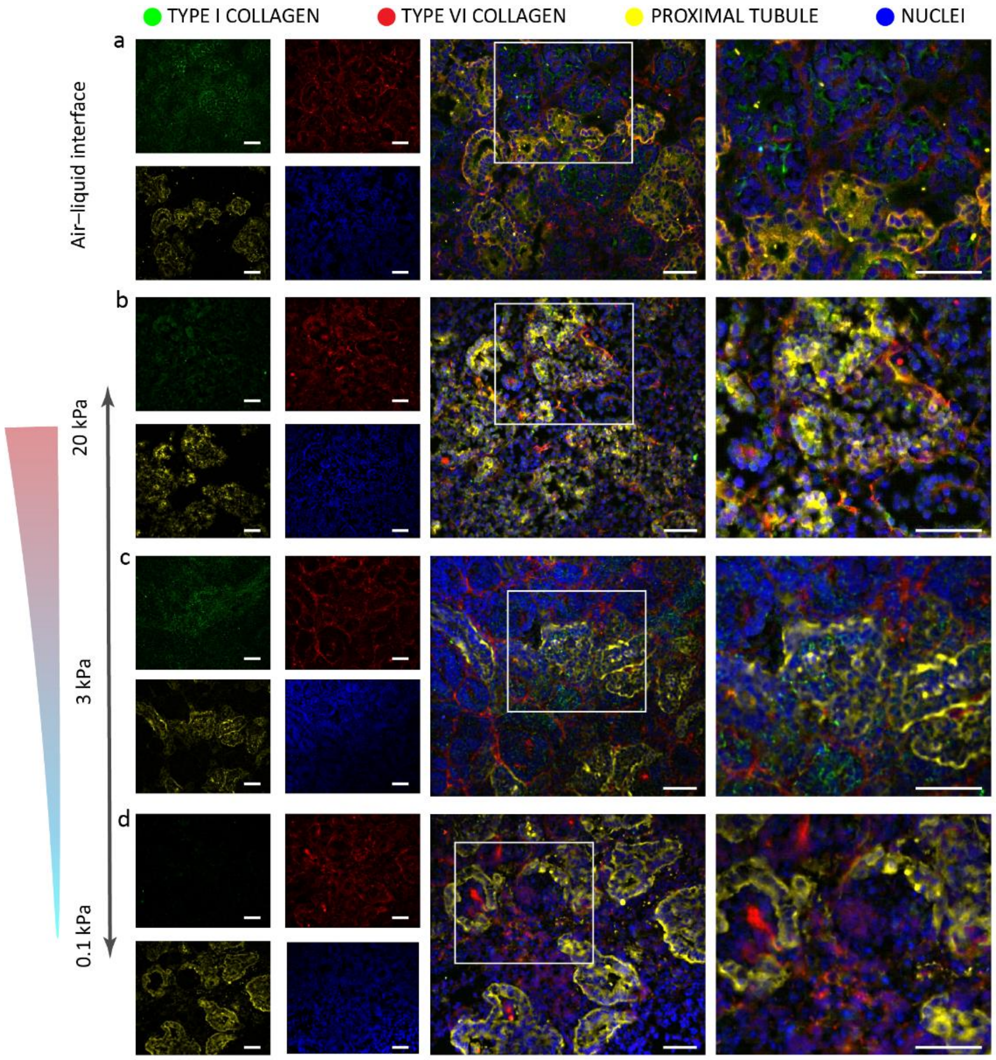

Figure S7. Reduced expression of collagen type 1a1 was observed in the encapsulated organoids. Immunohistochemistry for collagen type 1a1 (green), collagen type 6a1 (red), and LTL (yellow) on horizontally sectioned organoids encapsulated in the respective hydrogels (b–d) cultured on the air–liquid interface (a) at day 7+18. The reduced expression of type 1a1 collagen was observed in all encapsulated organoids (b–d) compared to organoids on the air–liquid interface (a). The white box denotes the area of interest enlarged in the respective right panel. Scale bars: 50 µm. Representative images of N=3 organoid batches with n=3 organoids per batch.

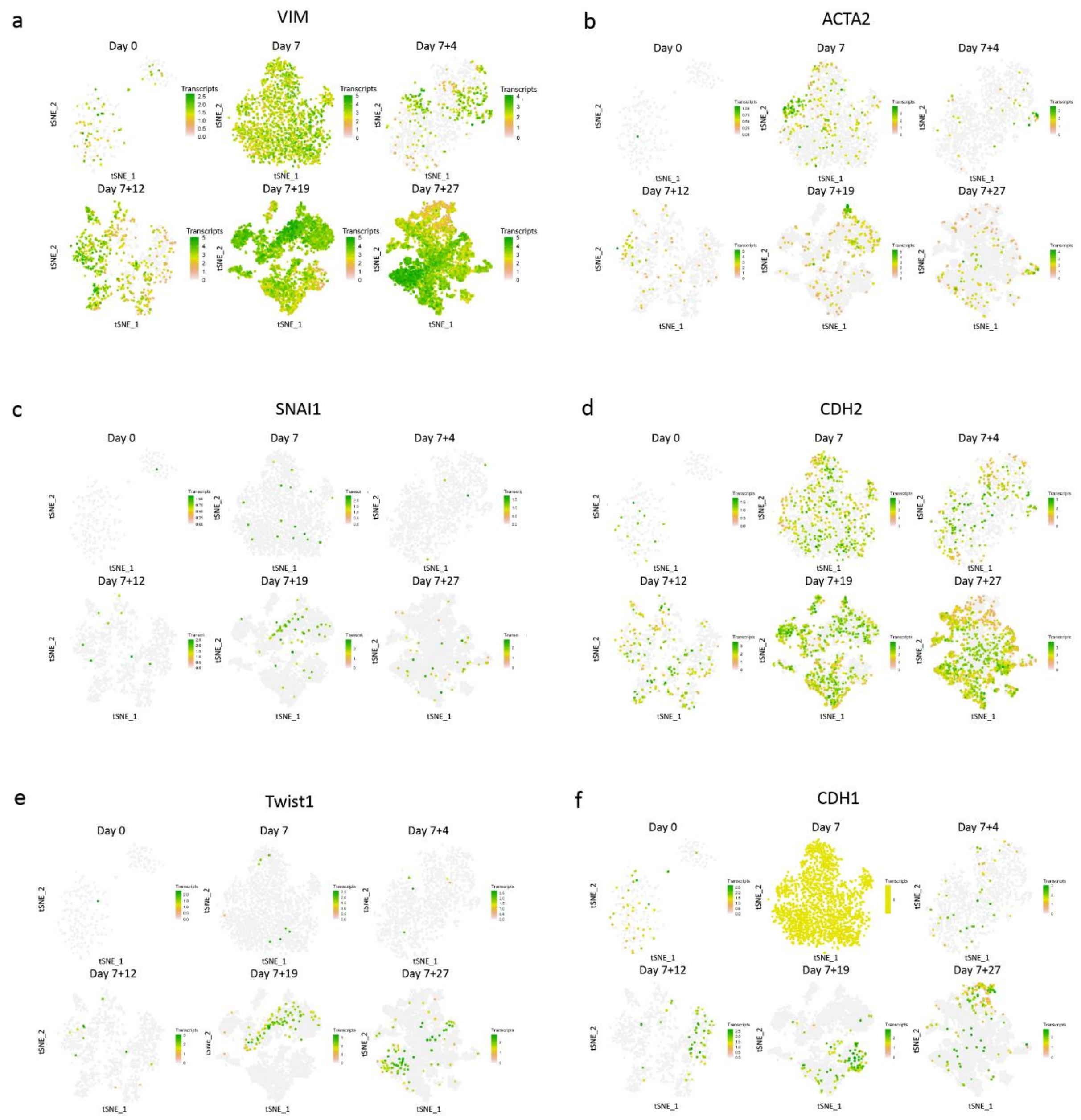

Figure S8. EMT-related gene expressions after prolonged air–liquid interface culture. Normalised gene expression of markers of interest shown in tSNE space of single-cell RNA (scRNA) sequencing data from the literature[18]. Cell populations expressing a) vimentin (VIM), b) actin alpha 2 smooth muscle (ACTA2), c) snail family transcriptional repressor 1 (SNAIL), d) N-cadherin (CDH2), and e) twist family bHLH transcription factor 1 (Twist1) increased in number when kidney organoids were cultured for 7+27 d. At the same time, cell populations expressing f) E-cadherin (CDH1) decreased during the culture.

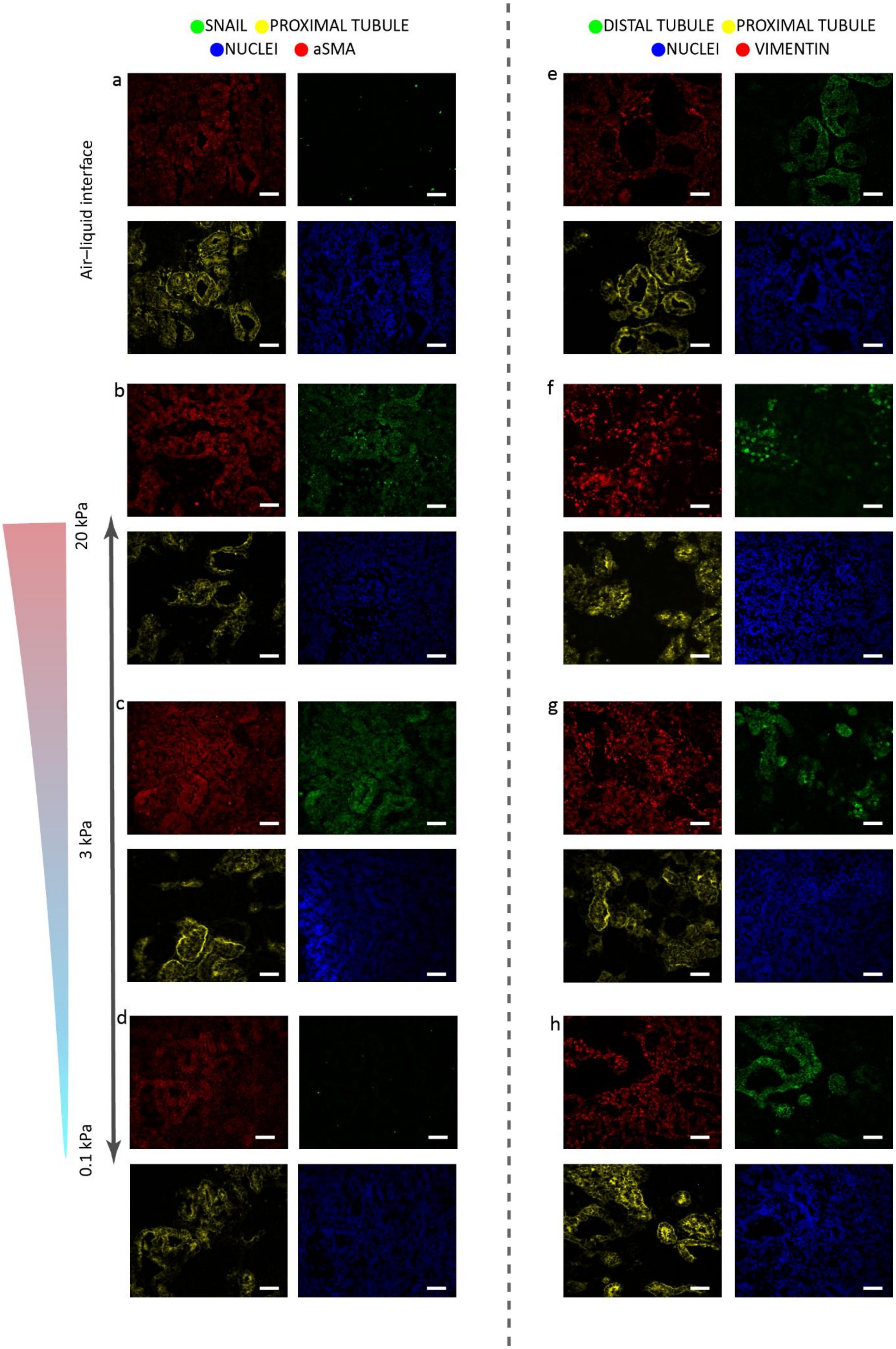

SNAIL
PROXIMAL TUBULE
NUCLEI
aSMA
DISTAL TUBULE
PROXIMAL TUBULE
NUCLEI
VIMENTIN
a
b
c
d
e
f
g
h
Air–liquid interface
20 kPa
3 kPa
0.1 kPa

Figure S9. Single channels of immunohistochemistry in **Figure 3b-i** of proximal tubules (LTL, lotus tetragonolobus lectin, in yellow), distal tubules (E-cadherin: ECAD, in green in far right column), SNAIL (in green in second column from left), aSMA (in red, far left column), and vimentin (in red, third column from left). DAPI staining (blue) for nuclei. Scale bars: 50 µm. Representative images of N=3 organoid batches with n=3 organoids per batch.

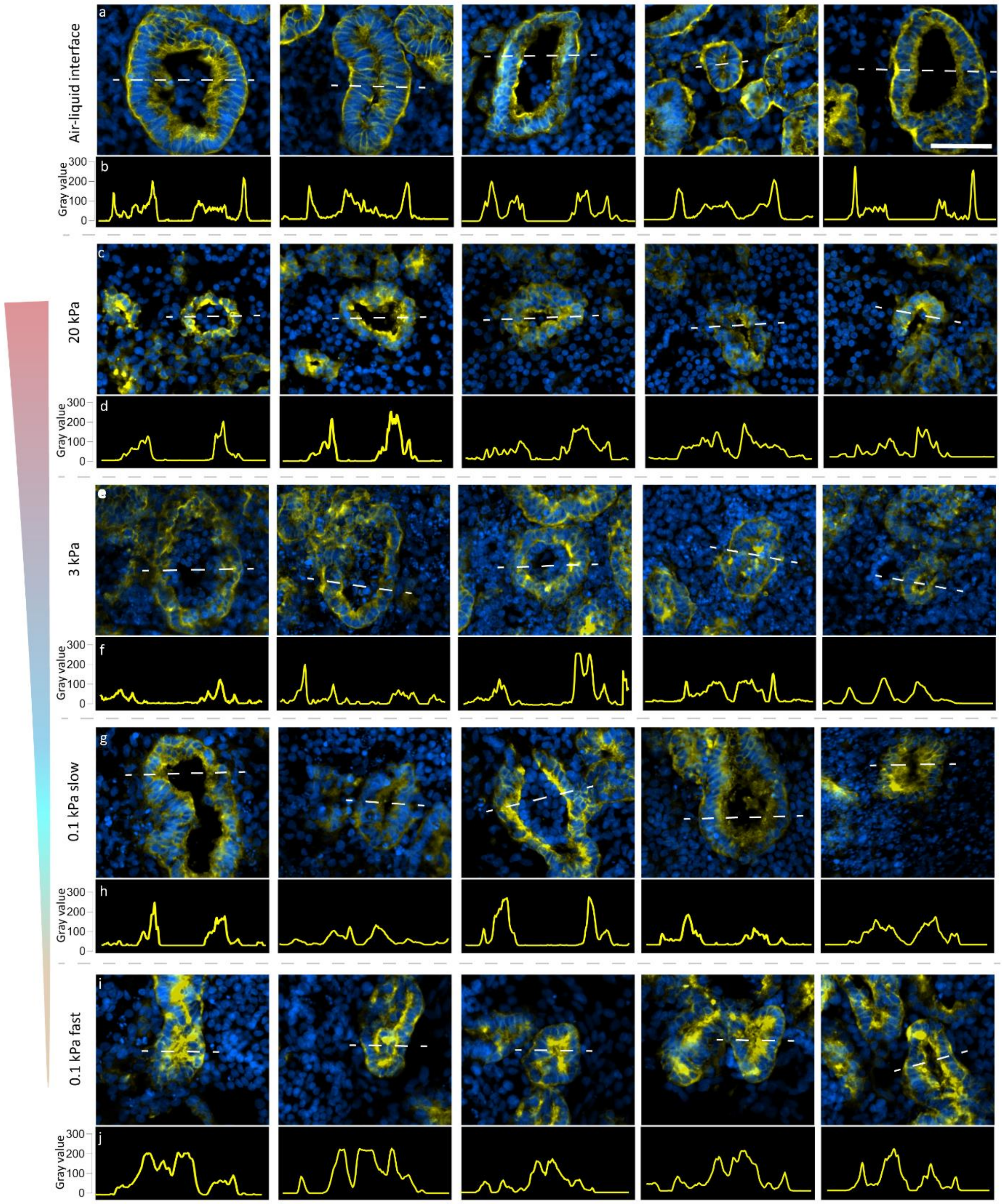

Figure S10. Lumen structures and apical enrichment. LTL staining was observed to be enriched in lumen structures of the organoids cultured in the 0.1 kPa hydrogels of fast (i-j) and slow (g-h) stress relaxing properties, compared to the air–liquid interface (a-b) while smaller lumen structures and less LTL enrichment were observed for the 3 kPa (e-f) and 20 kPa (c-d) hydrogels. Scale bar = 50 µm.

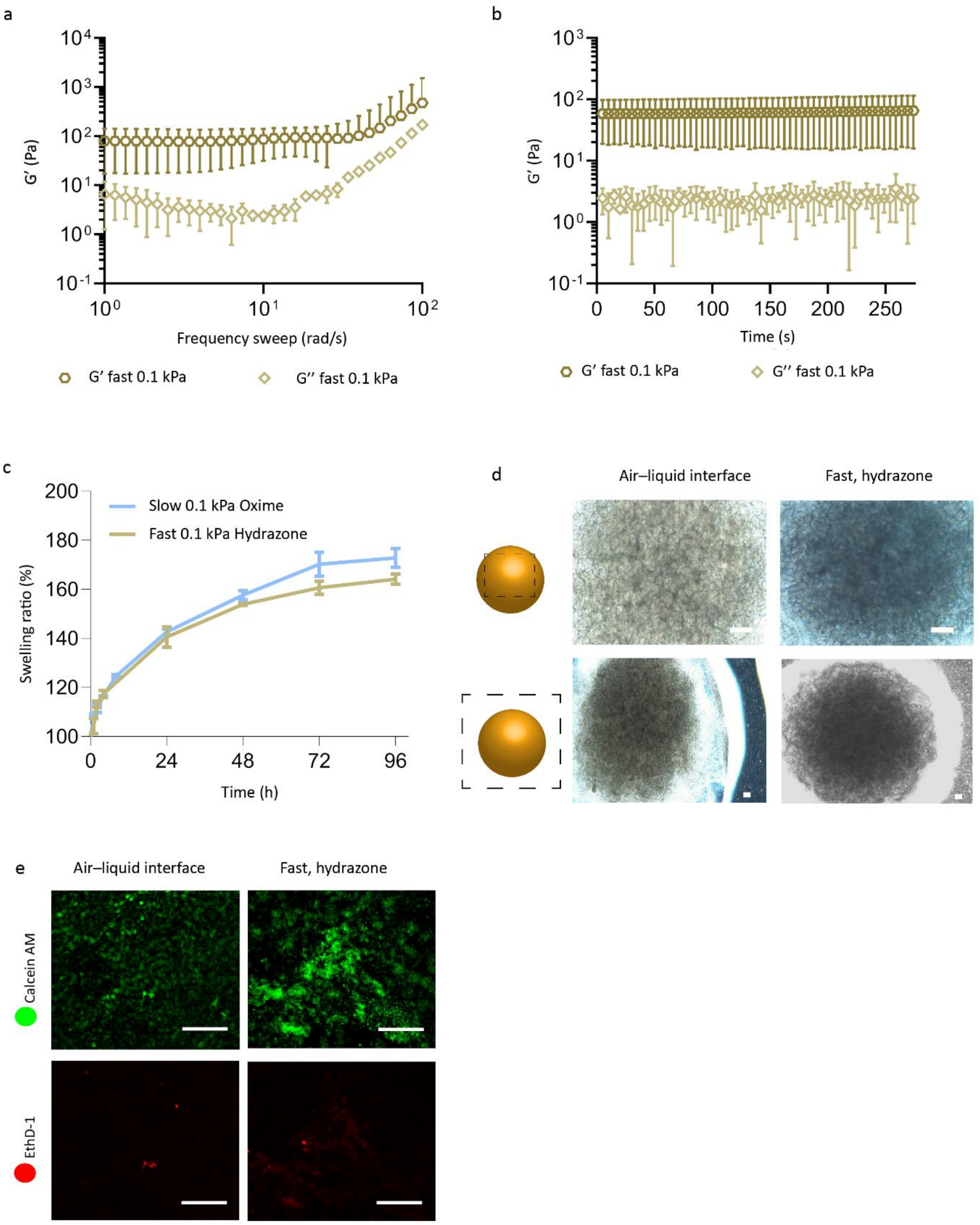

Figure S11. a) Frequency sweep data of the hydrogel (N=2) showed the hydrogel was not frequency dependent. b) Time sweep data of the hydrogel was used to determine the stiffness values of the hydrogel (N=2, Figure 1C). c) Swelling tests showed a swelling ratio of 165 % for the 0.1 kPa fast-relaxing hydrazone hydrogel after 96 h incubation, with no significant difference compared to the 172% swelling observed for the soft, slow-relaxing oxime cross-

linked hydrogels (unpaired t-test, *p*=0.0662, N=3). d) Bright field images of organoids in the fast hydrazone hydrogel were unchanged compared to the air–liquid interface organoids. e) Live/dead assay with calcein AM (live) and EthD-1 (red) showed no significant differences. Scale bars: 100 µm. Representative images of N=3 organoid batches with n=3 organoids per batch.

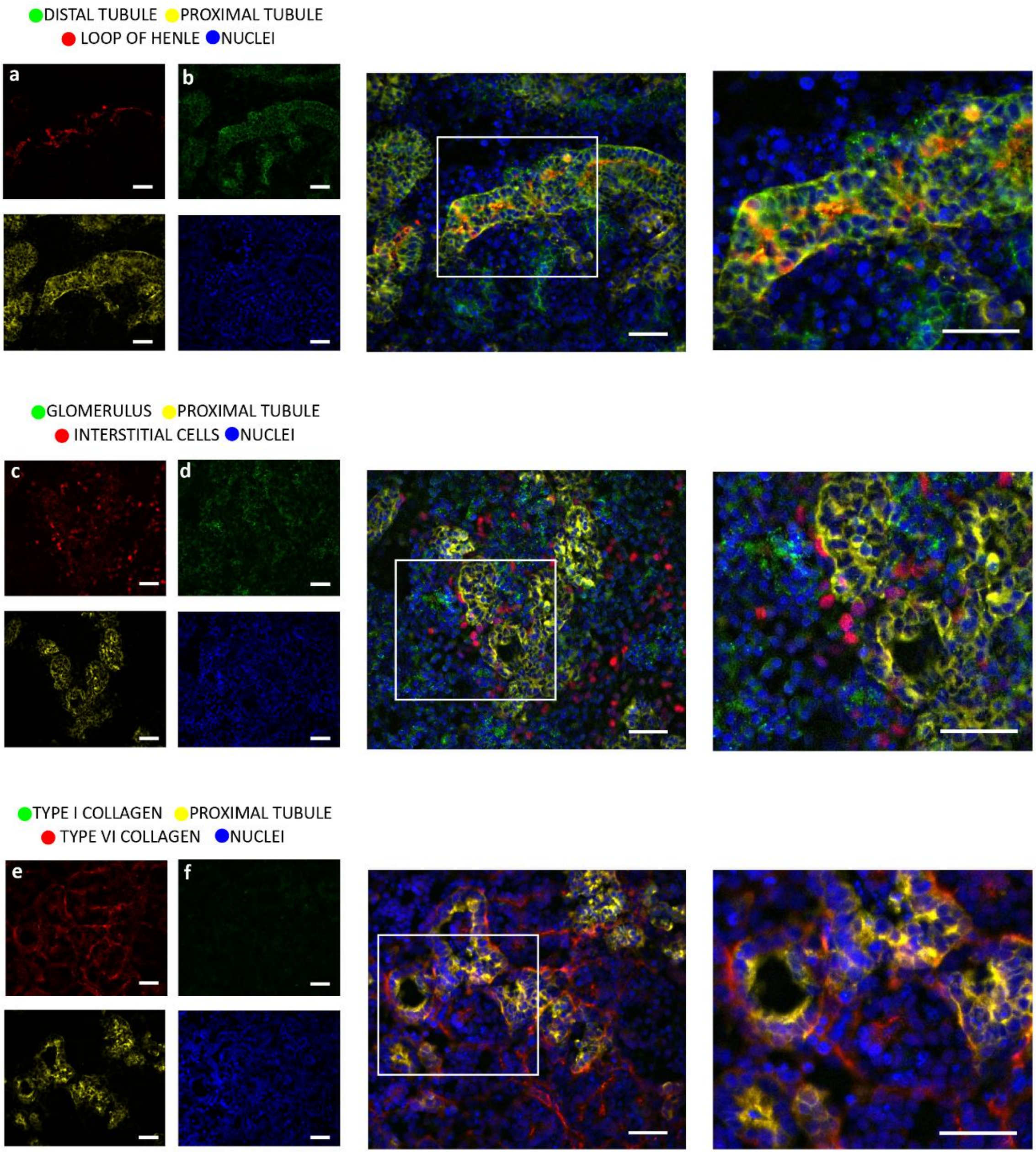

Figure S12. All renal cell types and reduced collagen type 1a1 were observed in the kidney organoids encapsulated in the 0.1 kPa, fast-relaxing hydrogel. Immunohistochemistry of kidney organoids encapsulated in the fast-relaxing hydrogel after 7+18 d. Staining was performed for the proximal tubules (LTL, lotus tetragonolobus lectin, in yellow), distal tubules (E-cadherin: ECAD, in green in a), loop of Henle (NKCC2: SLC12A1, in red in b), interstitial cells (homeobox protein Meis 1/2/3: MEIS1/2/3, in red in c), glomeruli (nephrin: NPHS1, in

green in d), type 1a1 collagen (green in e), and type 6a1 collagen (red in f). DAPI staining (blue) for nuclei. Single channels are shown in the two left columns; merged images in the two right columns. The white box denotes the area of interest enlarged in the respective panel to the right. Scale bars: 50 µm. Representative images of N=3 organoid batches with n=3 organoids per batch.

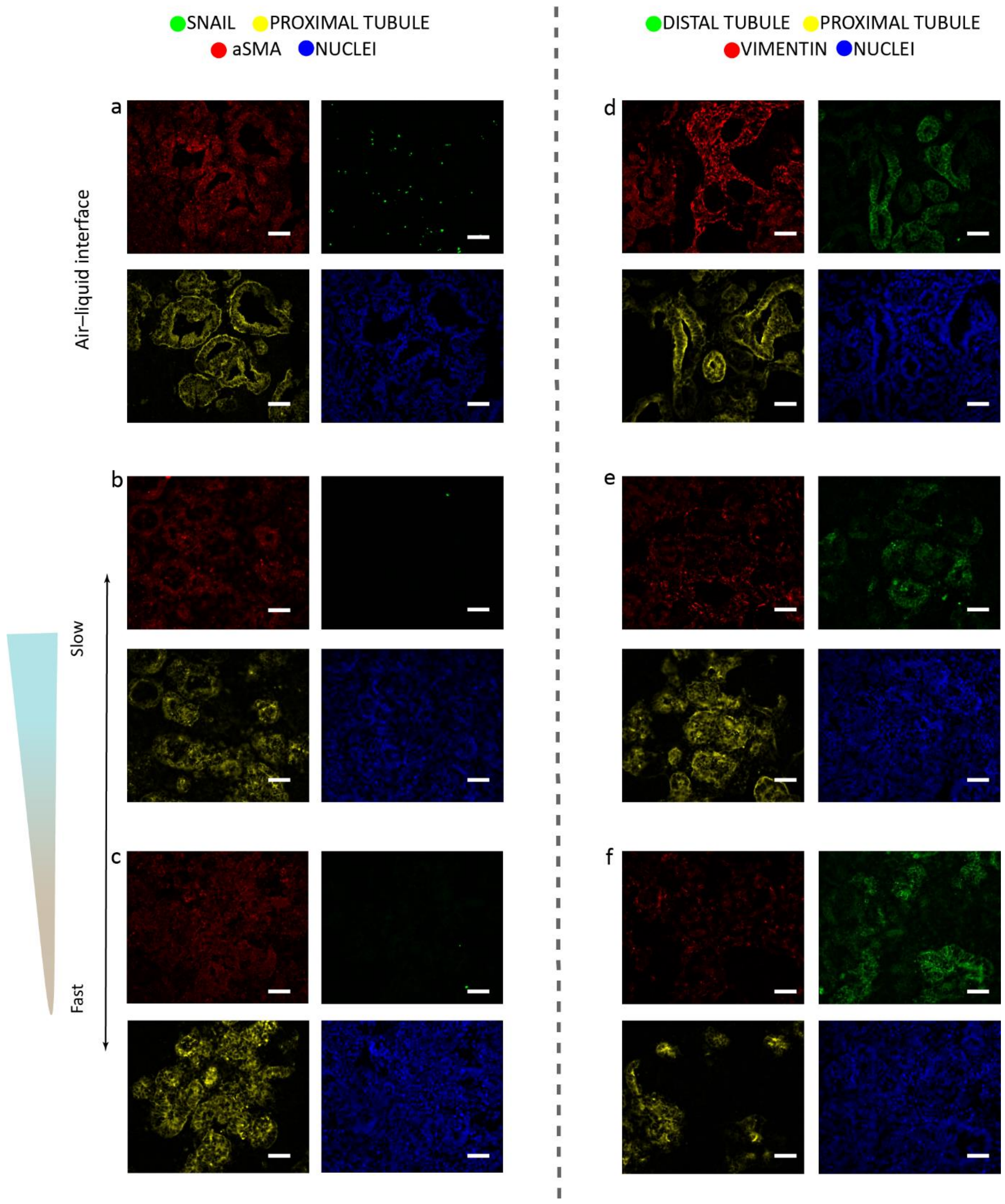

Figure S13. Single channels of immunohistochemistry in **Figure 4d-i** of proximal tubules (LTL, lotus tetragonolobus lectin, in yellow), distal tubules (E-cadherin: ECAD, in green in the far right column), SNAIL (green in the second column from the left), aSMA (red in the far left column), and vimentin (in red in the third column from left). DAPI staining (blue) for nuclei.

Scale bars: 50 μm. Representative images of N=3 organoid batches with n=3 organoids per batch.

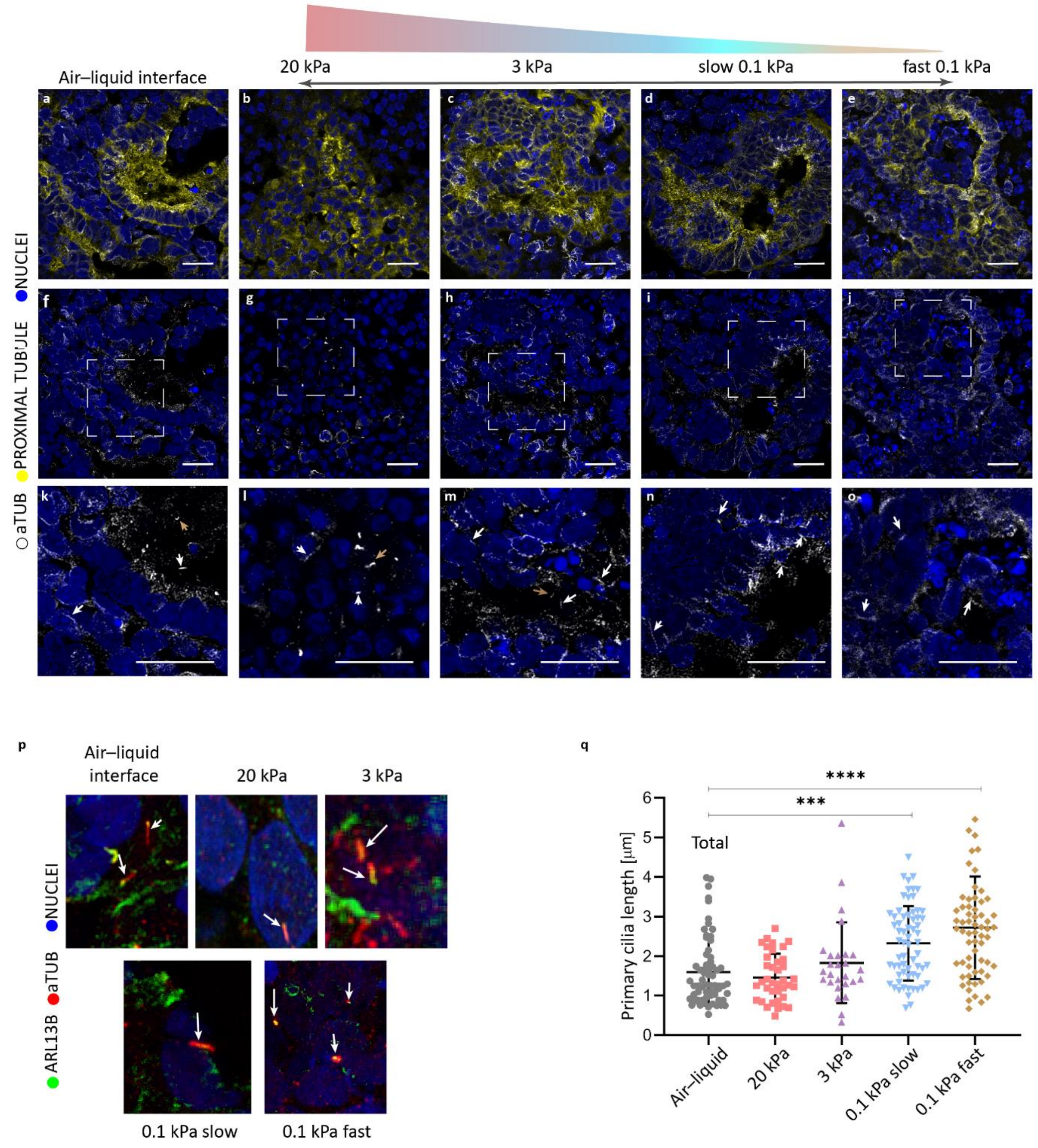

Figure S14. High resolution confocal images of primary cilia in a-f-k) air─liquid interface, b-g-l) 20 kPa, c-h-m) 3 kPa, d-i-n) 0.1 kPa slow-relaxation and e-j-o) 0.1 kPa fast relaxation. Scale bar: 20 μm. White boxes indicate area used for the zoomed in images k-o. Representative

images of N=3 organoid batches with n=3 organoids per batch. p) Costaining of primary cilia with aTUB and ARL13B. q) Primary cilia length in the different hydrogels comparted to the air–liquid interface. Significantly, longer cilium were observed in the softer hydrogels. (***<0.005 and ***<0.0005, one-way ANOVA).

Table S1. Molecular weight averages by GPC. Number average (M_n), weight average (M_w), and dispersity (M_w/M_n) for pure alginate and two oxidised alginate (Oxi-Alg) batches used for the study.

	M_n [kDa]	M_w [kDa]	M_w/M_n [Đ]
**Pure Alg**	130.7	316.2	2.46
**Oxi-Alg1**	28.3	69.6	2.46
**Oxi-Alg2**	83.5	185.3	2.21

Table S2. Volume, weights, and other properties of the prepared stock solutions and hydrogel solutions to form four different cross-linked alginate hydrogel systems used in these studies.

Stock solutions

Chemical	Weight [mg]	STEMdiff APEL2 or PBS [mL]	[mol/mL]	Wt%
**Oxi-alg-1 or -2**	165	2,75	-	6
**Oxime**	15	1.05	8.00×10^{-2}	-
**Hydrazone**	15	1.08	8.00×10^{-2}	-

Hydrogel solutions and properties for rheometry

Hydrogel	Oxi-alg (-1 or -2) [µL]	Crosslinker [µL]	PBS [µL]	Crosslinker [µM]	Oxi-alg [% w]
**0.1 kPa**	51.7 (1)	Oxime, 3.9	99.4	2.02	2
**3 kPa**	51.7 (1)	Oxime, 19.6	83.7	10.1	2
**20 kPa**	103.3 (1)	Oxime, 39.2	12.5	20.2	4
**Fast 0.1 kPa**	51.7 (2)	Hydrazone, 19.6	83.7	10.1	2

Hydrogel solutions and properties for organoid culture and swelling test

Hydrogel code	Oxi-alg (-1 or -2) [µL]	Crosslinker [µL]	STEMdiff APEL2 medium [µL]	Crosslinker [µM]	Oxi-alg [% w]	Stiffness [kPa]
**0.1 kPa**	166.7 (1)	Oxime, 12.6	320.7	2.02	2	0.08
**3 kPa**	166.7 (1)	Oxime, 63.2	270.2	10.1	2	3
**10 kPa**	333.3 (1)	Oxime, 126.3	40.4	20.2	4	20
**Fast 0.1 kPa**	166.7 (2)	Hydrazone, 63.2	320.7	10.2	2	0.1

Table S3. Set threshold boundaries set to measure grey intensities of single antibodies.

Antibody	Lower Threshold level	Upper Threshold Level
**aSMA**	19	65535
**ECAD**	15	65535
**LTL**	31	65535
**MEIS1/2/3**	12	65535
**NPHS1**	15	65535
**SLC12A1**	15	65535
**SNAIL**	33	65535
**Type I Collagen (COL1A1)**	10	65535
**Type VI Collagen (COL6A1)**	16	65535
**Vimentin (VIM)**	18	65535
